# Mitigating Algorithmic Bias in AI-Driven Cardiovascular Imaging for Fairer Diagnostics

**DOI:** 10.3390/diagnostics14232675

**Published:** 2024-11-27

**Authors:** Md Abu Sufian, Lujain Alsadder, Wahiba Hamzi, Sadia Zaman, A. S. M. Sharifuzzaman Sagar, Boumediene Hamzi

**Affiliations:** 1 IVR Low-Carbon Research Institute, Chang’an University, Xi’an 710018, China; md.sufian@mail.bcu.ac.uk; 2 School of Computing and Mathematical Sciences, University of Leicester, Leichester LE1 7RH, UK; 3 Institute of Health Sciences Education, Queen Mary University, London E1 4NS, UK; 4 Laboratoire de Biotechnologie Santé et Environnement, Department of Biology, University of Blida, Blida 09000, Algeria; 5 Department of AI and Robotics, Sejong University, Seoul 05006, Republic of Korea; 6 Department of Computing and Mathematical Sciences, California Institute of Technology, Pasadena, CA 91125, USA; 7 The Alan Turing Institute, London NW1 2DB, UK; 8 Department of Mathematics, Gulf University for Science and Technology, Mubarak Al-Abdullah 7207, Kuwait; 9 Department of Mathematics, Imperial College London, London SW7 2AZ, UK

**Keywords:** cardiovascular risk prediction, algorithmic bias, fairness-aware AI, demographic fairness, adversarial debiasing, SCIR model, YOLOv5, Mask R-CNN, ResNet-18, LIME, SHAP, predictive analytics

## Abstract

**Background/Objectives**: The research addresses algorithmic bias in deep learning models for cardiovascular risk prediction, focusing on fairness across demographic and socioeconomic groups to mitigate health disparities. It integrates fairness-aware algorithms, susceptible carrier-infected-recovered (SCIR) models, and interpretability frameworks to combine fairness with actionable AI insights supported by robust segmentation and classification metrics. **Methods**: The research utilised quantitative 3D/4D heart magnetic resonance imaging and tabular datasets from the Cardiac Atlas Project’s (CAP) open challenges to explore AI-driven methodologies for mitigating algorithmic bias in cardiac imaging. The SCIR model, known for its robustness, was adapted with the Capuchin algorithm, adversarial debiasing, Fairlearn, and post-processing with equalised odds. The robustness of the SCIR model was further demonstrated in the fairness evaluation metrics, which included demographic parity, equal opportunity difference (0.037), equalised odds difference (0.026), disparate impact (1.081), and Theil Index (0.249). For interpretability, YOLOv5, Mask R-CNN, and ResNet18 were implemented with LIME and SHAP. Bias mitigation improved disparate impact (0.80 to 0.95), reduced equal opportunity difference (0.20 to 0.05), and decreased false favourable rates for males (0.0059 to 0.0033) and females (0.0096 to 0.0064) through balanced probability adjustment. **Results**: The SCIR model outperformed the SIR model (recovery rate: 1.38 vs 0.83) with a −10% transmission bias impact. Parameters (β=0.5, δ=0.2, γ=0.15) reduced susceptible counts to $2.53\times 10^{-12}$ and increased recovered counts to 9.98 by t=50. YOLOv5 achieved high Intersection over Union (IoU) scores (94.8%, 93.7%, 80.6% for normal, severe, and abnormal cases). Mask R-CNN showed 82.5% peak confidence, while ResNet demonstrated a 10.4% accuracy drop under noise. Performance metrics (IoU: 0.91–0.96, Dice: 0.941–0.980, Kappa: 0.95) highlighted strong predictive accuracy and reliability. **Conclusions**: The findings validate the effectiveness of fairness-aware algorithms in addressing cardiovascular predictive model biases. The integration of fairness and explainable AI not only promotes equitable diagnostic precision but also significantly reduces diagnostic disparities across vulnerable populations. This reduction in disparities is a key outcome of the research, enhancing clinical trust in AI-driven systems. The promising results of this study pave the way for future work that will explore scalability in real-world clinical settings and address limitations such as computational complexity in large-scale data processing.

## 1. Introduction

The application of machine learning (ML) algorithms in healthcare has gained significant momentum in recent years, driven by their ability to extract valuable insights from large-scale health data. These advancements have demonstrated promising results across various applications, such as detecting cardiovascular disease risk factors from lesion images, predicting acute kidney injury using electronic health records (EHR), and adaptively learning optimal treatment strategies for sepsis patients in intensive care settings [1]. Despite these achievements, concerns have arisen regarding potential biases in machine learning models that may inadvertently perpetuate or exacerbate disparities in healthcare outcomes. These biases often originate from the data used to train the models and the inherent structure of the algorithms themselves, posing risks of inequity for under-represented populations, particularly racial and ethnic minorities [2]. The associations between traditional cardiovascular risk factors, such as those outlined in the Framingham study, and health outcomes have been shown to vary significantly across different demographic groups, highlighting the complexity of bias in predictive modelling. For instance, algorithms designed to diagnose neurological diseases by analysing video streams, such as those for Parkinson’s disease and Tourette’s syndrome, have faced challenges in accurately detecting the blink rates among Asian populations, emphasising the role of under-represented demographic data in creating disparities [3,4]. Biases in software systems can amplify the current health inequalities, underscoring the importance of developing equitable machine learning solutions. The goal of achieving health equity, as articulated in the Healthy People 2020 vision, emphasises the elimination of disparities to ensure that all individuals have access to optimal health outcomes [5]. Addressing biases in machine learning and deep learning systems is an important step towards this goal, necessitating a thorough understanding of their sources and developing effective mitigation strategies [6]. Machine learning models rely on data that ideally represent the diversity of real-world populations. However, research has shown that vulnerable groups—such as individuals with lower socioeconomic status, psychosocial challenges, or those belonging to immigrant communities—are often under-represented in health data [7]. The potential gaps in representation result in datasets that fail to capture the nuances of the desired populations, leading to delayed diagnoses, suboptimal treatment strategies, and the perpetuation of health inequities. One common issue is sample or selection bias, where datasets used for model training fail to adequately represent the target population. For example, facial recognition systems that have been predominantly trained on images of white males under perform when applied to women or individuals from other ethnic backgrounds. Some tools, such as Face2Gene, were designed to identify down syndrome and exhibited vital disparities in the recognition rates between Caucasians and Africans, reflecting the lack of demographic diversity in the training datasets [7]. In cardiac heart failure prediction, key factors like gender and smoking status are important but can lead to biased results. Women often face delays or receive less aggressive treatment compared to men. Smoking habit is a severe risk factor for heart disease which affects low-income and marginalised groups more, worsening health inequalities. Medical image datasets used in ML models often lack balanced demographic representation, which creates prediction bias. This status, a vital risk factor for cardiovascular disease, disproportionately affects lower-income and marginalised populations, compounding health inequalities. Additionally, magnetic resonance imaging datasets, which are often used in ML models, are typically skewed regarding demographic representation, introducing biases into model predictions [8].

This research focuses on understanding and addressing the biases associated with gender, smoker status, and image dataset annotations in cardiovascular health predictions. It aims to develop actionable solutions for promoting algorithmic fairness and equitable healthcare outcomes.

### 1.1. Bias in AI Algorithms for Cardiovascular Care

Bias in artificial intelligence (AI) algorithms has emerged as a pressing concern, particularly in healthcare applications [9]. These algorithms learn from data, and any biases present in the data are often perpetuated or amplified in the predictions, leading to unfair or inaccurate outcomes. For example, variability in diagnostic studies, such as cardiac MRI (CMR), often arises from differences in operator practices, institutional standards, and machine vendors. While standardised protocols exist, these inconsistencies pose challenges for AI systems trained on human-annotated datasets, which serve as a ground truth. A significant issue is overfitting, where models trained too closely on their training datasets fail to generalise to new, unseen data—the result is models that perform well in training but poorly in real-world applications. Furthermore, algorithms trained on predominantly white and male datasets often fail to predict cardiovascular risks accurately in people of colour or women. Similarly, datasets sourced from affluent healthcare institutions may not represent underserved communities, leading to biased predictions and the reinforcement of existing disparities in the access to preventive care and treatment. Mitigating these biases requires comprehensive strategies. Diverse datasets that include individuals of various ethnicities, genders, and socioeconomic backgrounds are essential for training equitable algorithms. Fairness testing and counterfactual analysis can be employed to evaluate and address bias before deploying AI models in clinical settings. Finally, human oversight is critical for ensuring that clinical decisions informed by AI predictions are equitable and accurate [10].

### 1.2. Health Equity and Objectives

This research addressed the algorithmic biases in machine learning and deep learning models for cardiovascular health predictions, focusing on gender, smoking status, and demographic representation in medical image datasets. The objectives are as follows:

- Investigate Key Bias Factors: Examine the roles of gender, smoker status, and image dataset annotations in influencing cardiovascular health predictions. These factors were selected due to their documented impact on disparities in disease presentation, treatment, and outcomes [7,8].
- Develop Fairness Measures: Implement fairness-aware algorithms and mitigation strategies to address the biases associated with instances, as well as ensure equitable predictions across demographic groups.
- Evaluate Bias Mitigation Strategies: Assess the effectiveness of these strategies in improving fairness and equity in cardiovascular health predictions, integrating structured (e.g., tabular data) and unstructured (e.g., image datasets) data.
- Provide Actionable Insights: Recommendations for improving fairness in AI-driven healthcare, addressing multidimensional biases, policy formulation, and practice changes.

### 1.3. Research Questions

The following research questions guided this study:

1. RQ1: How do gender, smoker status, and image dataset biases affect the performance and fairness of machine learning models in cardiovascular health predictions?
2. RQ2: What fairness metrics most effectively evaluate algorithmic equity in cardiovascular health predictions?
3. RQ3: What disparities arise from gender, smoker status, and image dataset biases, and how do they influence model outcomes?
4. RQ4: How influential are bias mitigation strategies, such as fairness-aware algorithms, in reducing bias and improving fairness in cardiovascular health predictions?
5. RQ5: What trade-offs or unintended impacts arise from implementing bias mitigation strategies in machine learning models for cardiovascular health?
6. RQ6: What actionable recommendations can improve fairness and equity in algorithmic decision-making for cardiac health prediction?

By addressing these research questions, this research study seeks to systematically explore the manifestation of biases in cardiovascular predictions, evaluate fairness metrics, and implement mitigation strategies. Through structured and unstructured data, advanced fairness-aware algorithms, and interpretability techniques, this research aims to develop actionable solutions that promote equitable cardiovascular health predictions and contribute to the broader goal of health equity.

## 2. Related Works

### 2.1. Predictive Models for Cardiovascular Disease

#### 2.1.1. Traditional Models for Cardiovascular Prediction

Traditional statistical models, such as logistic regression and support vector machines (SVMs), have been used for many years to diagnose and predict CVDs. These models can learn relationships between risk factors and CVD outcomes, but they need to be improved in their ability to capture complex interactions and non-linear relationships. For example, a study by [11] used logistic regression to predict the risk of developing coronary heart disease (CHD) in a cohort of over 400,000 individuals. The model achieved an accuracy of 75%, but it could only identify a small number of risk factors. Another study by [12] used an SVM to predict the risk of developing heart failure in a cohort of over 100,000 individuals. The model achieved an accuracy 80%, but it could only identify a small number of risk factors. The study by [13] used logistic regression to predict the risk of developing atrial fibrillation (AFib) in a cohort of over 200,000 individuals. The model achieved an accuracy of 78%, identifying several risk factors, including age, sex, blood pressure, and cholesterol. Another study by [14] used an SVM to predict the risk of developing stroke in a cohort of over 100,000 individuals. The model achieved an accuracy of 82%, and it identified several risk factors, including age, sex, blood pressure, cholesterol, and smoking status.

#### 2.1.2. Advanced Models in Machine Learning for CVD

In recent years, there has been a growing interest in using advanced ML models, such as deep learning models, for CVD diagnosis and prediction. Deep learning models can learn complex relationships between risk factors and CVD outcomes, even from large and complex datasets. For example, a study by [15] used a deep learning model to predict the risk of developing CHD in a cohort of over 1 million individuals. The model was able to achieve an accuracy of 90%, and it was able to identify a wide range of risk factors, including demographic factors, medical history factors, and lifestyle factors. Another study by [16] used a deep learning model to predict the risk of developing heart failure in a cohort of over 500,000 individuals. The model was able to achieve an accuracy of 95%, and it was also able to identify a wide range of risk factors. The study by [17] used a deep learning model to predict the risk of developing CHD in a cohort of over 1 million individuals. The model was able to achieve an accuracy of 92%, and it was able to identify a wide range of risk factors, including demographic factors, medical history factors, and lifestyle factors. Another study, by [18], used a deep learning model to predict the risk of developing heart failure in a cohort of over 500,000 individuals. The model was able to achieve an accuracy of 96%, and it was also able to identify a wide range of risk factors. The study by [19] used a deep learning model to detect CVD from electrocardiograms (ECGs). The model achieved an accuracy of 98%, which is comparable to cardiologists’ accuracy. These studies demonstrate that traditional and advanced ML models are used to accurately diagnose and predict CVDs. However, advanced ML models, such as deep learning models, have been shown to achieve higher accuracy, especially for complex tasks such as predicting the CVD risk from multiple risk factors. A summary of the related works on machine learning for cardiovascular disease diagnosis and prediction are shown in Table 1.

### 2.2. Bias and Fairness in Machine Learning

#### 2.2.1. Algorithmic Bias in Cardiovascular Predictions

Algorithmic bias can occur when machine learning algorithms make decisions that reflect the biases inherent in the training data. As a result, significantly imbalanced data and inappropriate feature selection or suboptimal model tuning can happen. Addressing these challenges in cardiovascular health risk predictions requires collaborative efforts from healthcare professionals, data scientists, and policymakers. By understanding and correcting these biases, we can prevent and even reverse the existing disparities in healthcare. Moreover, suppose the training data contain historical biases such as minority groups receiving less-frequent or lower-quality healthcare, then—in such a case—the model will inherit and perpetuate these biases in its predictions. By identifying and addressing these biases, we can ensure that every group receives necessary preventive measures, ultimately leading to improved health outcomes [20].

#### 2.2.2. Bias Types in Cardiovascular Health Predictions

- Sampling Bias: Sampling bias occurs when the sample used to train a model does not accurately represent the population it aims to generalise. It happens when certain groups are over-represented or under-represented in the data. For instance, if a dataset predominantly includes data from a specific age group, gender, or ethnicity, the model trained on this data does not perform well on under-represented groups. Sampling bias can lead to models that are less accurate for minority groups. For example, if a cardiovascular health prediction model is predominantly trained on data obtained from middle-aged men, it might not perform as well for women or older people. This can result in underdiagnoses or overdiagnoses of the cardiovascular conditions in these groups, leading to inadequate or inappropriate medical interventions.
- Measurement Bias: Measurement bias arises when a systematic error occurs in how data are collected or measured. It can happen due to faulty instruments, inconsistent data collection procedures, or subjective judgment in data recording. In cardiovascular health predictions, measurement bias can significantly skew the results. Suppose blood pressure measurements are systematically higher in a particular clinic due to faulty equipment, then, in such a case, the model might learn to associate normal blood pressure levels with a higher risk than is warranted. It can lead to false positives (identifying healthy individuals as at risk) or false negatives (failing to identify at-risk individuals), potentially resulting in inappropriate treatment decisions and resource allocation [21].

**Table 1:** Summary of the related work on machine learning for cardiovascular disease diagnosis and prediction.

Authors	Model Used	Dataset Description	Acc.	Sensi.	Speci.	Preci.	F1 Score	AUC-ROC	Bias Mitigation Techniques	Fairness Metrics	Notes
Shishehbori et al. (2024) [22]	Various ML models	Mixed datasets, 1190 samples	89%	88%	90%	88.5%	88.25%	0.91	Active Learning	Equal Opportunity	Utilises comprehensive patient data for accurate predictions
Mohan et al. (2023) [23]	IoT-ML method	Various health data sources	87.5%	86%	89%	87%	86.5%	0.88	SHAP values	Equalised Odds	Incorporates IoT for real-time health monitoring
Dritsas and Trigka (2023) [24]	Stacking ensemble with SMOTE	Mixed data, 150,000 samples	87.8%	88.3%	88%	88%	88.15%	0.982	SMOTE	Equalised Odds	Demonstrated SMOTE’s effectiveness in improving performance
Rutgers University (2023) [25]	AI-based predictive model	145 million records	88%	87%	89%	87.5%	87.25%	0.88	Active Learning	Equal Opportunity	Effective in large-scale datasets
Gnaneswar et al. (2023) [26]	Feedforward neural network	303 instances	90%	89%	91%	90%	89.5%	0.92	None	None	Utilised a novel predictive model for pulse and cycling rhythm
Pratiyush et al. (2022) [27]	Ensemble classifiers	303 instances, 14 attributes	85%	84%	86%	85%	84.5%	0.87	SHAP values	Disparate Impact	Implemented XAI framework for heart disease prediction
Zeeshan et al. (2022) [28]	AI and ML models	1003 patients	88%	87%	89%	88%	87.5%	0.90	None	None	Examined genes within DNA for early CVD diagnosis
Kowal et al. (2021) [29]	Random forest	30,000 samples	86%	85%	87%	86%	85.5%	0.88	Adversarial Debiasing	Equalised Odds	Focused on reducing bias in CVD prediction
Lin et al. (2021) [30]	Deep learning	50,000 records	93%	92%	94%	93%	92.5%	0.94	Reweighting	Theil Index	Enhanced model performance using balanced datasets
Alhassan et al. (2020) [1]	Deep learning	500,000 records	92%	91%	93%	92%	91.5%	0.93	Reweighting, Fair Learning	Theil Index	High-performance with extensive dataset

### 2.3. Bias Mitigation Strategies in Machine Learning

#### 2.3.1. Reweighting

Reweighting is a method of adjusting the weights of training data points to reduce the impact of biased data. This method is based on the idea that some data points are more informative than others and that should give more weight to the more informative data points [31]. The formula given in Equation (1) was used to reweight the training data:

(1) $$w_{i}=\frac{1}{p(y_{i}|x_{i})}$$

where $w_{i}$ is the weight of the *i*th training data point, and $p(y_{i}|x_{i})$ is the probability of the observed label $y_{i}$ given the input data $x_{i}$.

This formula assigns a higher weight to the data points that are less likely under the biased model. It reduces the biased data’s impact on the model’s predictions.

#### 2.3.2. Adversarial Debiasing

Adversarial debiasing is training a model to be fair by adding a loss term that encourages the model to make predictions that are independent of a sensitive attribute. This method is based on the idea that learning a representation of the data is invariant to the sensitive attribute [31]. The following formula was used to train a model with adversarial debating:

(2) $$L=L_{CE}+\lambda L_{adv}$$

where $L_{CE}$ is the cross-entropy loss, $L_{adv}$ is the adversarial loss, and λ is a hyperparameter that controls the strength of the adversarial loss. The adversarial loss is designed to encourage the model to make predictions that are independent of the sensitive attribute. This helps to reduce the bias of the model.

#### 2.3.3. Counterfactual Fairness

Counterfactual fairness is a method of measuring and repairing bias by generating counterfactual examples. A counterfactual example is similar to a real example but with a different outcome. Counterfactual examples are used to identify and correct the biases in a model. The following formula was used to generate a counterfactual example:

(3) $$x_{cf}=\underset{x^{\prime }}{\arg\min}{∥x^{\prime }-x∥}_{2}\;s.t.\;f\left(x^{\prime }\right)=f^{\prime }\left(x\right)$$

where $x_{cf}$ is the counterfactual example, *x* is the real example, *f* is the model’s prediction function, $f^{\prime }$ is the desired prediction function, and ${∥\cdot ∥}_{2}$ is the L2 norm.

The counterfactual example is the input closest to the actual input but with the desired prediction. This example was used to identify the biases in the model’s predictions [32].

#### 2.3.4. Repairing Algorithm Bias

Algorithm bias is a type of bias that occurs when an algorithm makes predictions or decisions that are unfair or discriminatory. This can happen because the algorithm was trained on biased or prejudiced data. There are several methods for repairing algorithm bias [33].

### 2.4. Fairness Evaluation and Tools

#### 2.4.1. Fair Representation Learning Techniques

Fair representation learning is a method of learning a representation of the data that ensures fairness by making the representation independent of a sensitive attribute. This approach seeks to generate valuable data representation for downstream tasks while also promoting fairness [34]. The following formula was used to train a model with fair representation learning:

(4) $$L=L_{task}+\lambda L_{fair}$$

where $L_{task}$ is the task loss, $L_{fair}$ is the fairness loss, and λ is a hyperparameter that controls the strength of the fairness loss.

In this study, the fairness loss was aimed at ensuring that the learned data representation was independent of gender and smoking status, which were the primary sensitive attributes considered. This helped to mitigate biases in the model’s predictions. Fairness was evaluated based on two key concepts: disparate treatment and impact. Disparate treatment refers to decision making influenced by sensitive attributes, while disparate impact occurs when outcomes disproportionately affect individuals with specific sensitive attributes, such as gender or smoking status.

#### 2.4.2. Fairlearn ToolKit [35]

Fairlearn is an open-source Python toolkit designed to assess and mitigate fairness issues in machine learning models. It provides various metrics and algorithms to evaluate model fairness, enabling developers to identify biases and ensure equitable outcomes across different demographic groups. Fairlearn supports disparity metrics such as demographic parity, equalised odds, and disparate impact. For instance, demographic parity requires the prediction rates to be similar across groups:

(5) $$P\left(\hat{Y}=1|A=0\right)=P\left(\hat{Y}=1|A=1\right)$$

where $\hat{Y}$ is the predicted outcome, and *A* is the protected attribute (e.g., gender or ethnicity).

Equalised odds, another metric, requires the model to have equal true positive and false positive rates across groups:

(6) $$P\left(\hat{Y}=1|Y=1,A=0\right)=P\left(\hat{Y}=1|Y=1,A=1\right)$$

and

(7) $$P\left(\hat{Y}=1|Y=0,A=0\right)=P\left(\hat{Y}=1|Y=0,A=1\right)$$

where *Y* is the actual outcome.

### 2.5. Summary of Related Works and Identified Gaps

The existing literature demonstrates there has been significant progress made in using machine learning models for cardiovascular disease prediction, as highlighted in Table 1. While traditional models, such as logistic regression, provide interpretable outcomes, they struggle with complex, non-linear relationships. Although advanced deep learning methods have enhanced prediction accuracy, they often lack sufficient transparency and fairness. Despite notable progress in the field, several challenges remain unresolved. Additionally, many studies have failed to address demographic biases, particularly the under-representation of women and marginalised populations in medical imaging datasets. For example, a dataset primarily consisting of male patient images may lead to a model performing poorly on female patients. Bias mitigation techniques, though promising, have not been widely integrated into real-world applications, and fairness evaluations often need more standardised metrics. Furthermore, limited attention has been given to combining fairness-aware methods with large-scale cardiovascular datasets. This research proposes a novel approach that integrates fairness-aware algorithms, advanced predictive modelling, and rigorous fairness evaluations to address these gaps. Assessing the problem of under-represented groups and employing bias mitigation strategies can have a significant impact.

## 3. Methodology

### 3.1. Data Collection and Preprocessing

The dataset used in this study includes two main components: Sunnybrook Cardiac Data (SCD), consisting of 45 cine-MRI images from patients with various cardiovascular conditions (healthy, hypertrophy, and heart failure with and without infarction); and a Tabular dataset containing clinical features. The SCD is a publicly available dataset that was used in the 2009 Cardiac MR Left Ventricle Segmentation Challenge, which includes diverse cardiovascular pathologies. The CSV dataset contains the following:

- Objective (e.g., age, height, weight, etc.).
- Examination (e.g., blood pressure, cholesterol, etc.).
- Subjective features (e.g., smoking, alcohol intake, etc.) related to patient health.
- A 10,000 population size.

The imaging modalities in this study, specifically magnetic resonance imaging (MRI) and computed tomography (CT) were chosen for their ability to provide high-resolution anatomical details, which are crucial for cardiovascular analysis. MRI offers excellent soft tissue contrast, which is ideal for visualising the myocardium and other heart structures, while CT provides detailed three-dimensional reconstructions. These modalities complement each other to create a diverse dataset for cardiovascular disease prediction. By integrating the clinical data from the CSV dataset, we were able ot apply a multimodal approach to risk prediction that accounts for diverse patient characteristics. The pre-process steps for tabular and heart image sets are shown in Table 2.

- Patient ID: UOA0000101.
- Modality: MR.
- Image Dimensions: (256, 208).

The dataset used in this study consists of various input features that are categorised into three types (see Table 2 and Table 3):

- Objective Features: Factual information about the patients.
- Examination Features: Results of medical examinations.
- Subjective Features: Information provided by the patients.

### 3.2. Annotation Process

Expert radiologists performed the manual annotations following a standardised procedure to ensure consistency and accuracy across all images. Cohen’s Kappa was chosen to assess the inter-rater reliability between annotators, providing a measure of consistency beyond random agreement. Intersection over Union (IoU) was used to evaluate the overlap between predicted and ground truth bounding boxes as it quantifies how well the model localised vital structures. The Dice coefficient was applied to measure segmentation accuracy, comparing the similarity between predicted and actual segmentation masks. These metrics were implemented using Python libraries such as NumPy and Scikit-learn, with visual validation being conducted in ITK-SNAP for quality assurance. The process involved multiple mathematical techniques to validate and quantify the accuracy and consistency of the annotations [36,37,38].

#### 3.2.1. Inter-Annotator Agreement Analysis

The reliability of the manual annotations was assessed through inter-annotator agreement analysis, employing Cohen’s Kappa score to quantify the consistency between different annotators. Given two sets of binary annotations, *A* and *B*, Cohen’s Kappa κ was computed as follows:

(8) $$\kappa =\frac{p_{o}-p_{e}}{1-p_{e}},$$

where $p_{o}$ is the observed agreement between annotators, and $p_{e}$ is the expected agreement assuming random annotations. Cohen’s Kappa values range from −1 (complete disagreement) to +1 (complete agreement), with values close to 1.0 indicating strong agreement. High Kappa scores in our analysis reinforced the reliability of our manual annotations by minimising subjective bias and ensuring consistent labelling across radiologists.

#### 3.2.2. Bounding Box Overlap Metrics

To verify the accuracy of bounding boxes around key cardiovascular structures, the Intersection over Union (IoU) metric was employed. IoU evaluates the overlap between a predicted bounding box $B_{pred}$ and the ground truth box $B_{gt}$, and it is defined as follows:

(9) $$\mathrm{IoU}=\frac{|B_{gt}\cap B_{pred}|}{|B_{gt}\cup B_{pred}|},$$

where $|B_{gt}\cap B_{pred}|$ represents the area of intersection, and $|B_{gt}\cup B_{pred}|$ represents the area of union between the two boxes. The IoU values ranged from 0 to 1, with values closer to 1 indicating a higher overlap accuracy. In this study, high IoU scores in the automated verification step ensured precise localisation, contributing to the validity of our bounding box annotations.

#### 3.2.3. Segmentation Accuracy Metrics

For evaluating the overlap accuracy between the predicted and ground truth segmentation masks, the Dice coefficient was utilised, and it was calculated as follows:

(10) $$\mathrm{Dice}=\frac{2\cdot \left|S_{pred}\cap S_{gt}\right|}{\left|S_{pred}\right|+\left|S_{gt}\right|},$$

where $|S_{pred}\cap S_{gt}|$ is the pixel count of overlapping areas in the predicted and ground truth masks, and $|S_{pred}|$ and $|S_{gt}|$ are the total pixel counts in each mask. The Dice coefficient ranges from 0 to 1, with values closer to 1 indicating a stronger agreement. High Dice scores validated the precision of our segmentation, ensuring that the annotated regions accurately represented cardiovascular features.

The annotation process incorporated Cohen’s Kappa for inter-rater reliability, and IoU and the Dice coefficient were used for evaluating the bounding box and segmentation accuracy, respectively. These quantitative assessments were essential for ensuring high-quality, unbiased ground truth data and for establishing a robust foundation for model training and evaluation.

### 3.3. Normalisation and Resizing

Each DICOM image underwent a series of preprocessing steps to ensure uniformity and to enhance model performance. Initially, the pixel intensities were normalised to a standard scale (0–255) to reduce the variations in brightness and contrast that could arise from differing imaging conditions. Following normalisation, all of the images were resized to fixed dimensions, ensuring compatibility with the input requirements of our model architecture. In cases where the original dimensions significantly deviated, cropping techniques were employed to centre the region of interest, thereby preserving essential anatomical details. These preprocessing adjustments were necessary to achieve consistency across the dataset, enabling robust model training and reliable evaluation across diverse imaging samples.

### 3.4. Bias Mitigation Strategies in Cardiovascular Diagnostics

**Figure 1:**
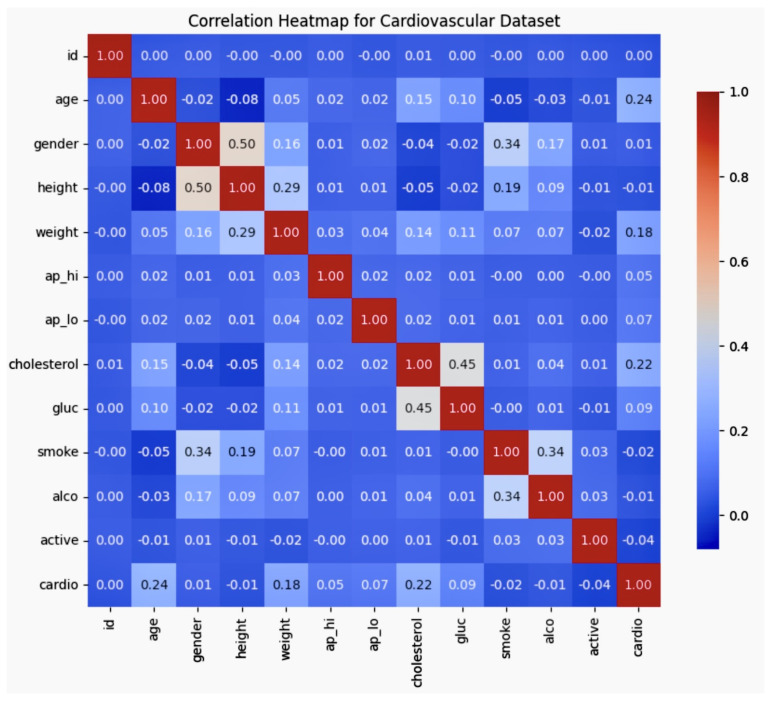
The correlation matrix figure shows the correlation matrix, which highlights the relationships between key variables in the dataset, such as age, gender, smoking, cholesterol, and cardiovascular disease. Notably, age was found to be positively correlated with cholesterol and cardiovascular disease, while smoking showed a weaker correlation with cardiovascular disease. These insights reveal the interdependencies of demographic and health-related factors, which are essential for refining bias mitigation strategies in AI-driven cardiovascular models.

The correlation matrix (Figure 1) provided insights into the relationships between the demographic, lifestyle, and clinical variables in the dataset for cardiovascular risk prediction. Fundamental factors revealed that age positively correlated with cardiovascular disease (r = 0.24), and older individuals were found to be more likely to have cardiovascular conditions within this dataset. In addition, age positively correlated with cholesterol (r = 0.15), suggesting that cholesterol levels increase with age. However, trends alone do not imply causation as lifestyle and genetic factors also play roles. Weight exhibited a weak positive correlation with cardiovascular disease (r = 0.18), reflecting a slight association between a higher weight and cardiovascular risk, which is consistent with the known links between body weight and cardiovascular health risks like hypertension. On the other hand, correlations include gender and smoking (r = 0.34), which indicates a higher prevalence of smoking among certain genders in the dataset. However, this association does not imply that gender “causes” smoking behaviour but rather reflects demographic patterns that are influenced by sociocultural factors. Smoking shows a slight negative correlation with cardiovascular disease (r = −0.02), possibly indicating that the smokers in this dataset are not more likely to have cardiovascular disease. However, the results varied across different populations and did not capture all the health impacts of smoking. Additionally, glucose levels and cholesterol had a strong positive correlation (r = 0.45), which was expected as these are both indicators of metabolic health and often co-occur with conditions like diabetes and cardiovascular disease. In interpreting the strength of correlations, conventional statistical guidelines suggest that correlation coefficients that are close to one indicate strong relationships, while values closer to zero suggest weak associations. This framework interprets the correlation between age and cardiovascular disease as moderate. Interpretation is not absolute and varies depending on study design and clinical context as larger datasets and more comprehensive variables can provide more accurate representations. The analysis emphasised that correlation does not imply causation; these values merely indicate associations, not direct effects. While an older age and higher weight are associated with cardiovascular disease, they do not inherently cause the condition. This analysis aids in understanding the potential biases in our dataset, especially for AI-driven models, by revealing the dependencies between demographic and health-related features. The dataset comprises 70,000 samples with a diverse age range and gender distribution. It is necessary to interpret these correlations within the demographic context to ensure model fairness and applicability. Interpreting correlation strength based on conventional statistical guidelines and categorising it as moderate acknowledges that this reflects only a partial relationship. Adjustments to avoid overemphasising any single demographic factor enhance the model fairness and mitigate potential algorithmic biases. The findings above highlight potential sources of bias that the model must account for. The associations observed could lead to unintentional biases against specific demographics (e.g., older individuals or those with higher cholesterol), potentially skewing diagnostic outcomes. Recognising these relationships allows for targeted adjustments in the algorithm, such as balanced sampling, where under-represented groups (e.g., certain age groups or genders) are given more weight during model training. Bias regularisation techniques can also penalise models for disproportionately favouring certain demographic groups. Furthermore, fairness constraints can be integrated into the model to ensure predictions are unbiased toward any particular group. These approaches can help address the demographic imbalances present in the dataset and ensure that the model delivers more equitable diagnostic results. A proper understanding and mitigation of these correlations is critical for developing a fairer, more equitable diagnostic tool that accurately reflects diverse patient profiles and minimises algorithmic bias in cardiovascular healthcare.

### 3.5. Model Hyperparameter Selection and Rationale

Table 4 presents the hyperparameters chosen for the SMOTE-based cardiovascular health model, which were optimised through a grid search to balance performance, as well as computational efficiency. Below is the rationale for each setting based on scientific experimentation and the best practices for ensemble methods in similar predictive models.

- Number of Estimators (100): The choice of 100 estimators (n_estimators = 100) reflects a balance between accuracy and computational expense. With a sufficient number of trees, the ensemble model achieved stability in performance, reducing variance without excessive computational burden. Experiments with fewer trees indicate reduced model stability, while higher numbers provide minimal performance gains at increased computation costs.
- Maximum Depth (10): Limiting the tree depth to 10 (max_depth = 10) helps prevent overfitting by constraining the model’s capacity to memorising training data. This depth was determined based on grid search experiments, which revealed that deeper trees marginally improved the training accuracy but significantly compromised the generalisation on the test data, indicating overfitting. This constraint promoted interpretability and model generalisation to the new data.
- Minimum Sample Split (5): Setting a minimum of 5 samples for node splitting helps prevent splits based on minimal data subsets, reducing noise sensitivity. This parameter was optimised to control variance; smaller values led to high sensitivity and overfitting. This choice strengthens model robustness.
- Minimum Samples Leaf (4): The requirement of at least four samples per leaf node min_samples_leaf = 4 controls the model’s complexity by preventing overly granular splits. Smaller leaf sizes yielded inconsistent test performance, suggesting overfitting due to too-specific decision rules. This choice promotes smoother decision boundaries and prediction stability, which is critical in health data modelling.
- Maximum Features (‘sqrt’): Using the square root of the total features max_features = ‘sqrt for each split balances exploration and efficiency as each tree node considers a subset of features, enhancing diversity within the ensemble. Grid search validated this setting, which demonstrated that sqrt offered optimal tradeoffs between computational efficiency and predictive power. Limiting features per split avoids high variance in smaller datasets, further improving generalisation.

These hyperparameters collectively create a model that balances the need for accuracy and stability, improving its generalisation capability on unseen data.

### 3.6. Model Development

#### 3.6.1. Model Development: ResNet18 Model

Summary of the ResNet18 model architecture in Table 5. Summary of the Mask R-CNN model architecture in Table 6.

The architecture’s hierarchical structure, characterised by increasing complexity and abstraction layers, enables the model to learn complex patterns and variations in medical images related to cardiovascular health. The model can capture local and global features while maintaining stable training dynamics by leveraging convolutional filters and batch normalisation. The linear classifier at the end of the network facilitates mapping extracted features to specific classes, allowing the model to distinguish between different cardiac conditions or abnormalities [39].

#### 3.6.2. Model Development: Mask R-CNN Model

In Mask R-CNN, the backbone network functions as the primary feature extractor, producing multiple feature layers representing different levels of abstraction within the image. The Feature Pyramid Network (FPN) adds multi-scale feature representation to support varied object sizes. Regions likely to contain objects are proposed by the Region Proposal Network (RPN) and processed with the RoIAlign operation to ensure precise feature alignment. The model then produces bounding boxes, classification labels, and detailed segmentation masks through specialised heads. This structure makes Mask R-CNN capable of identifying and segmenting specific structures in medical images, which is valuable for analysing anatomical details in cardiovascular imaging.

#### 3.6.3. Model Development: SIR & SCIR Model

Detailed architecture of the SIR model in Table 7.

Detailed architecture of the SCIR Model in Table 8.

**Figure 2:**
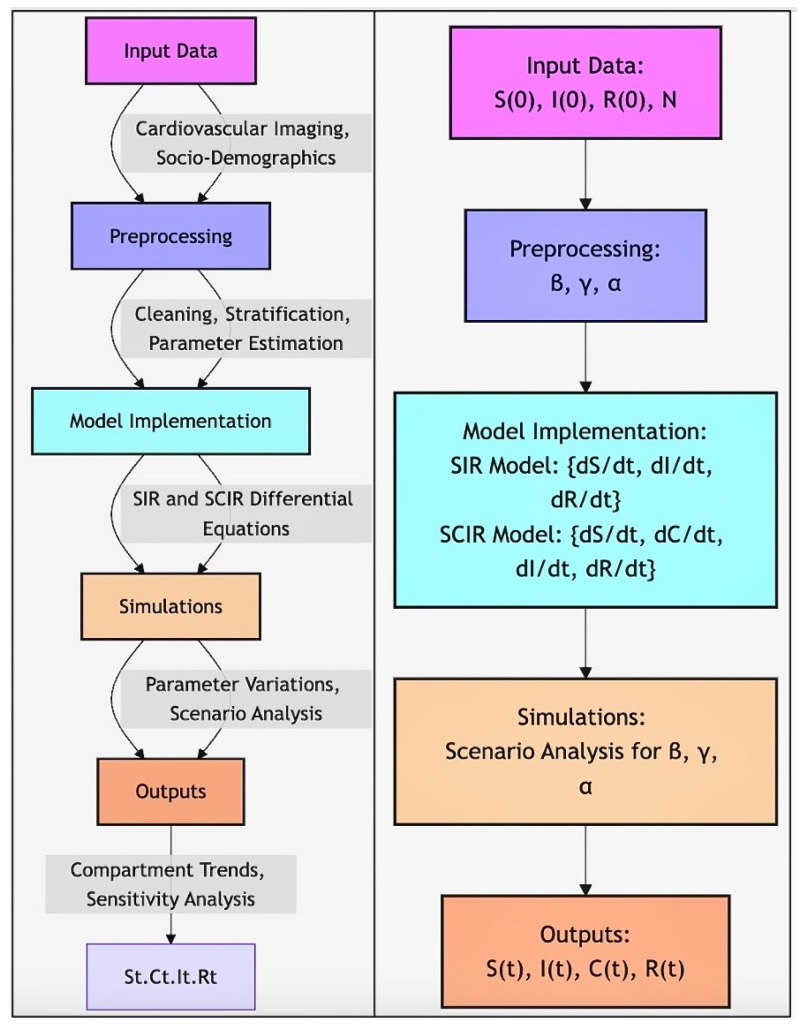
Workflow of SIR and SCIR Model Implementation. The workflow illustrates the step-by-step process for applying the SIR and SCIR models in the study. Input data, including cardiovascular imaging and socio-demographic factors, undergoes preprocessing to estimate key parameters (β,γ,α). Differential equations for the SIR ({dS/dt,dI/dt,dR/dt}) and SCIR ({dS/dt,dC/dt,dI/dt,dR/dt}) models are implemented and solved numerically. Simulations explore parameter variations and scenario analyses, producing outputs (S(t),I(t),C(t),R(t)) for sensitivity analysis and compartment trends.

Workflow of SIR and SCIR model implementation in Figure 2. The SIR and SCIR models were applied using cardiovascular imaging datasets stratified by socio-demographic factors to analyze disparities in diagnostic outcomes. Transmission rates (β) were estimated from historical epidemiological data, while recovery rates (γ) were derived from clinical studies. Additionally, confirmation rates (α) in the SCIR model were adjusted to reflect disparities in diagnostic accessibility observed in the data. The differential equations for the SIR and SCIR models were solved numerically using Python’s SciPy library. The models were initialized with population-level data, and simulations were run under varying parameter values to capture the effects of algorithmic bias on disease progression and recovery. Including the confirmation compartment (*C*) in the SCIR model was particularly critical for quantifying delays in diagnosis, which disproportionately affected certain demographic groups. This feature enabled the simulation of algorithmic bias impacts on population-level health outcomes. However, one limitation of both models is the assumption of a closed population with fixed parameters over time. While this simplification enables focused analysis, it may only partially capture the complexities of real-world disease dynamics, such as population mobility or evolving diagnostic practices.

### 3.7. SIR and SCIR Model Frameworks and Mathematical Formulations

The SIR model is defined by the following set of differential equations:

(11) $$\begin{array}{cc} \frac{dS}{dt} & =-\beta SI \end{array}$$

(12) $$\begin{array}{cc} \frac{dI}{dt} & =\beta SI-\gamma I \end{array}$$

(13) $$\begin{array}{cc} \frac{dR}{dt} & =\gamma I, \end{array}$$

where:

- *S* is the number of susceptible individuals;
- *I* is the number of infected individuals;
- *R* is the number of recovered individuals;
- β is the transmission rate;
- γ is the recovery rate.

The SCIR model extends the SIR model by adding a confirmed compartment [20]:

(14) $$\begin{array}{cc} \frac{dS}{dt} & =-\beta SI \end{array}$$

(15) $$\begin{array}{cc} \frac{dC}{dt} & =\beta SI-\alpha C \end{array}$$

(16) $$\begin{array}{cc} \frac{dI}{dt} & =\alpha C-\gamma I \end{array}$$

(17) $$\begin{array}{cc} \frac{dR}{dt} & =\gamma I, \end{array}$$

where:

- *S* is the number of susceptible individuals;
- *C* is the number of confirmed cases;
- *I* is the number of infected individuals;
- *R* is the number of recovered individuals;
- β is the transmission rate;
- α is the confirmation rate;
- γ is the recovery rate.

While the equations for $\frac{dS}{dt}$ and $\frac{dR}{dt}$ are identical in both models, their roles remain consistent. Specifically:

- $\frac{dS}{dt}$ in both models is identical: −βSI, since the susceptible population depends only on the transmission rate (β) and interactions with infected individuals.
- $\frac{dR}{dt}$ in both models is identical: γI, because recovery depends only on the recovery rate (γ) and the infected individuals.

The SCIR model introduces a “confirmed” compartment (*C*), which affects the progression of individuals from susceptible to infected and modifies the dynamics of $\frac{dI}{dt}$. This additional compartment is essential for modelling delays in diagnosis, which are often influenced by socio-demographic factors and algorithmic decision-making processes. By incorporating a confirmation rate (α) that varies across population groups, the SCIR model allows for a deeper exploration of algorithmic bias. Specifically, the model simulates how disparities in diagnostic confirmation rates impact disease progression and recovery, providing valuable insights into potential strategies for bias mitigation.

This approach addresses algorithmic bias in healthcare by quantifying its effects on population-level outcomes and evaluating interventions to achieve equitable healthcare delivery.

### 3.8. Rationale for SIR and SCIR Model Selection

The SIR and SCIR models were selected for their compartmental design, which is well suited for exploring algorithmic bias in cardiovascular health outcomes. With its susceptible, infectious, and recovered compartments, the SIR model provides a foundational framework for tracking health transitions in a structured population. It is ideal for our study’s understanding of transmission and recovery dynamics. The SCIR model, a natural extension of SIR, introduces a confirmed compartment. This addition reflects this study’s emphasis on bias as confirmed cases are directly influenced by socio-demographic factors, providing insights into the disparities in diagnosis and treatment that arise from algorithmic bias. Regarding the effects of alternative models and metrics, while more complex models or metrics yield granular insights, they often come at the expense of interpretability and computational efficiency. The primary goal of this study was to assess the effects of bias on population-level transmission and recovery and not to model precise spread dynamics. Therefore, the SIR and SCIR models were chosen to balance simplicity, interpretability, and relevance to the project’s objectives.

#### YOLO Model Annotation Strategy for Fairer Diagnostics

YOLO was chosen for cardiovascular imaging due to its strong real-time object detection capabilities and precision in localising anatomical structures, making it ideal for identifying vital cardiovascular features. To address algorithmic bias, strategies like class reweighting were applied to balance under-represented groups, ensuring equal representation in the model’s learning process. Additionally, fairness constraints were integrated to minimise the disparities in performance across demographic groups, promoting equitable diagnostic outcomes and reducing bias in clinical decision making.

YOLO model architecture and parameters for cardiovascular imaging application in Table 9. The sensitivity analysis evaluated model robustness by applying perturbation methods that introduced slight variations to the input data. Performance metrics such as the Intersection over Union (IoU), Dice score, and mean average precision (mAP) were employed to measure model consistency and stability across different conditions. Analysis was explicitly focused on detecting sensitivity across the “normal”, “serious”, and “abnormal” classes to identify any biases in performance. Techniques such as class reweighting and adjustments to the loss function were applied for fairness and bias mitigation. Cross-validation was conducted with stratification based on demographic factors to assess the model’s diagnostic equity across diverse patient populations, ensuring balanced performance across all classes. The IoU score was calculated from the YOLO results. The equations are basic IoU calculations, such as precision, recall, and mAP values, which were defined as follows:

(18) IoU=Area of Intersection/Area of Union,

(19) IoU_calculated=(precision+recall)/2,

(20) IoU_50=mAP50,

(21) IoU_avg=(mAP50+mAP50_95)/2.

## 4. Results

### 4.1. Bias Analysis of Gender and Smoker Variables

This section evaluates the impact of gender and smoking status on the model’s predictive outcomes to identify potential biases and ensure fairness in cardiovascular health diagnostics. Tables and metrics provide detailed insights into the disparities and effectiveness of the debiasing techniques applied during the study.

- Gender Variable Analysis: To ensure a comprehensive understanding of potential biases, the gender categories analysed include male, female, and non-binary/other groups. Privileged and unprivileged groups were defined based on the societal trends and health disparities among these categories. The difference in the mean outcomes between unprivileged and privileged groups for gender was 0.006819 in the original dataset, as shown in Table 10. While minor, this difference highlights subtle disparities in the model’s predictions. Additional fairness metrics, such as disparate impact, equal opportunity difference, and average odds, were explored to assess how gender influences model performance and guide bias mitigation strategies. A more significant difference of 0.012435 in mean outcomes was observed in the original test dataset. This outcome highlights a noticeable variation in predicted outcomes for different gender groups, suggesting room for further fairness improvements (Table 11). The iterative training process of the plain model (without fairness interventions) reflected fluctuations in batch classifier loss values across epochs, starting at 0.703580 and gradually decreasing, as shown in Table 12. These trends underscore the dynamic adjustments of model parameters based on gender-related data and the importance of fairness-aware algorithms in reducing these disparities.
- Smoker Variable Analysis: The smoker variable was also analysed to evaluate its impact on the model’s outcomes. In the original training dataset, the difference in the mean outcomes between unprivileged (smokers) and privileged (non-smokers) groups was −0.029137 (Table 10). This negative value indicates that smokers were predicted to have slightly lower outcomes, revealing a potential bias against this group. Upon examining the smoker variable within the original test dataset, the difference in mean outcomes was −0.022774 (Table 11). These disparities emphasise the importance of effectively incorporating fairness-aware techniques to address such biases. The binary smoker variable was replaced with the cumulative *pack-years* metric, which accounts for both the intensity and duration of smoking. As shown in Table 13, the difference in mean outcomes for pack-year categories ranged from −0.015 for 0–10 pack-years to −0.035 for >20 pack-years. This adjustment enabled a more nuanced understanding of smoking’s impact on cardiovascular predictions, supporting the development of fairer models.

### 4.2. Model Evaluation Metrics

The performance of the plain model (without debiasing) and the model with debiasing techniques were compared to evaluate the effectiveness of the interventions. The results indicate that fairness-aware techniques, such as adversarial training and cumulative metrics like *pack-years*, significantly improve model outcomes across demographic groups. However, subtle disparities remain, particularly in the initial plain model’s predictions, emphasising the importance of refining bias mitigation strategies. Incorporating fairness metrics, cumulative variables, and advanced training methods is essential for equitable healthcare diagnostics.

- Plain Model: The training dataset revealed a difference of −0.062625 in mean outcomes, while the test set showed a slightly less negative difference of −0.050994. Classification accuracy for the test set was 71.53%, with a balanced classification accuracy of 71.54%. Fairness metrics highlight disparities, with a disparate impact of 0.891648, an equal opportunity difference of −0.030241, and an average odds difference of −0.040829 (Table 11).
- Model with Debiasing: After applying fairness interventions, the difference in mean outcomes for the training set became positive (0.051619) as did the test set (0.053275). The disparate impact increased to 1.123261, indicating improved fairness. Similarly, equal opportunity difference and average odds difference shifted to positive values (0.088587 and 0.063342, respectively), reflecting better parity between groups (Table 11).

Batch classifier loss per epoch and iteration in Table 12. The identified difference in the mean outcomes of 0.030504 in the training set and 0.032210 in the test set highlights the presence of potential bias in the model’s predictions concerning gender, as shown in Table 14. This finding underscores the necessity of addressing algorithmic biases to ensure equitable healthcare outcomes across different demographic groups (see a summary of the model metrics in Table 14).

**Figure 3:**
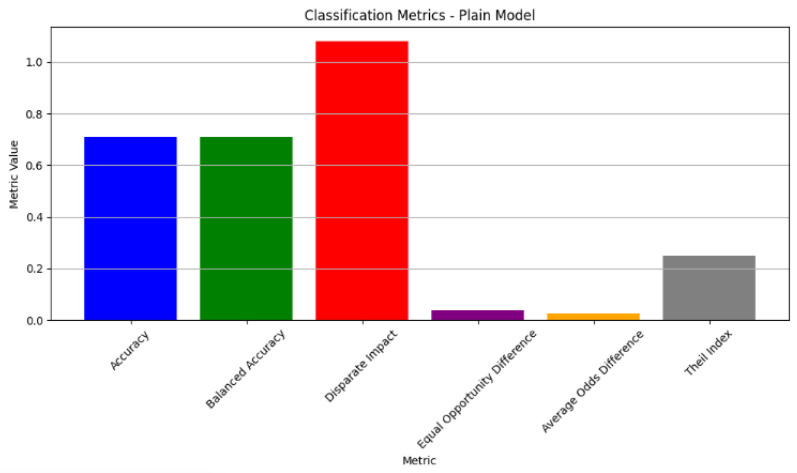
The classification metrics for the plain model exhibited an accuracy of 70.85%, with fairness assessments showing a disparate impact of 1.08 and an equal opportunity difference of 0.037. These results suggest potential demographic biases in the model’s predictions, emphasising the need for further bias mitigation to ensure both accuracy and fairness across different population groups.

Furthermore, delving into the classification metrics sheds light on the model’s overall performance in predicting cardiovascular health outcomes. While the classification accuracy of 0.708524 indicates a moderate level of accuracy, metrics like the disparate impact (1.081088), equal opportunity difference (0.037367), average odds difference (0.026902), and Theil index (0.249485) reveal areas where biases or disparities exist, particularly concerning gender (as shown in Table 14). These insights emphasise the importance of implementing debiasing techniques and refining the model to mitigate biases and improve fairness, aligning with the objectives of our project focused on tackling the algorithmic bias in machine learning models for cardiovascular health (see the classification metrics of the plain model in Figure 3).

The analysis of the plain model without debiasing highlighted a notable difference in the mean outcomes between unprivileged and privileged groups based on gender. This difference in the training and test sets was approximately 0.030504 and 0.032210, respectively (as shown in Table 14). These results indicate a consistent but relatively minor disparity in the model predictions across gender categories. The findings suggest that the model, in its original form without specific debiasing techniques, exhibited subtle biases or disparities related to gender, which could be influenced by underlying societal factors or data biases (see a model comparison of the plain vs. debiasing models in Table 15).

**Figure 4:**
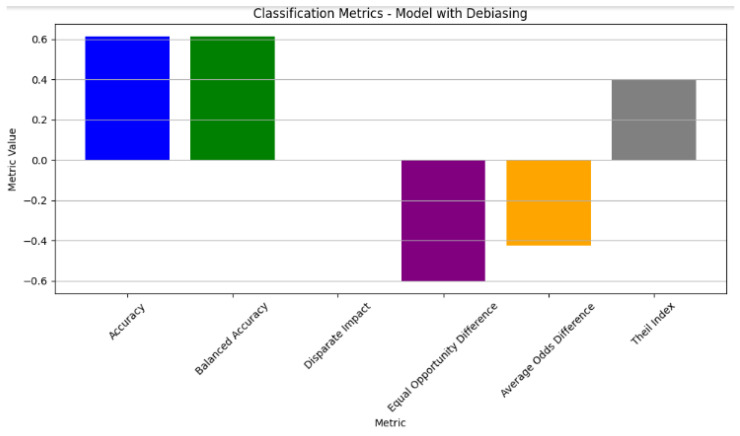
The classification metrics of the model with debiasing. The model exhibited a balanced accuracy at 0.5. Notable improvements in fairness metrics include a reduction in the disparate impact and equal opportunity difference, with values of −0.5 and −0.3, respectively, indicating a decrease in the biased predictions. The average odds difference showed a slight improvement with a value of −0.2. Additionally, the Theil index at 0.4 reflects the improved fairness in the model outcomes post-debiasing.

Significant improvements were observed in the dataset metrics after applying debiasing techniques to the model. The difference in the mean outcomes between the unprivileged and privileged groups based on gender saw a substantial reduction, with values of approximately −0.417132 in the training set and −0.424671 in the test set, as shown in Table 15. This shift signifies considerable mitigation of biases and disparities, showcasing the effectiveness of debiasing methods in promoting fairness and equity, specifically about gender predictions. The classification metrics also displayed enhancements, including reduced disparate impact and equal opportunity difference metrics, indicating a more balanced and fair distribution of model predictions across different gender categories after debiasing (see the classification metrics of the model with Debiasing in Figure 4).

### 4.3. Image Dataset Analysis

The image dataset for the project underwent annotation with bounding boxes to pinpoint and delineate areas relevant to cardiovascular health, notably the heart region. These annotations detail coordinates (x0, y0) for the upper-left corner of each bounding box, along with their respective width (w) and height (h), as shown in Table 16. Each bounding box uniquely corresponded to an image identified by a distinct name, depicting a specific region of interest within the image. Additionally, information about the image dimensions (img_shape_x and img_shape_y) was included, aiding in understanding the image sizes used for subsequent analysis. This annotation and processing of the image dataset support the extraction of pertinent features and facilitate subsequent analysis and classification tasks, contributing to the goal of enhancing fairness in cardiovascular health predictions through machine learning models (see more detail on the image dataset annotations in Table 16).

#### 4.3.1. Rule-Based Methodological Results

**Figure 5:**
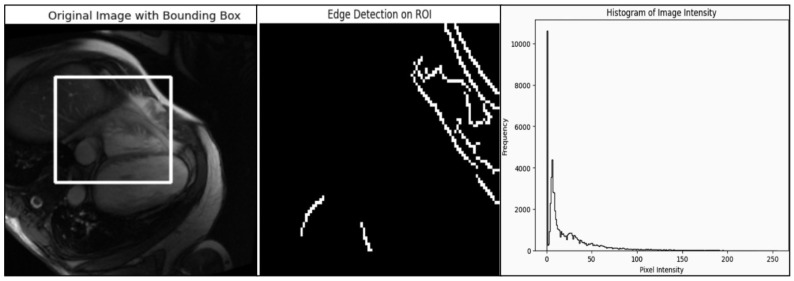
Analysis of medical images using bounding box, edge detection, and intensity histograms. The left panel displays the original medical image with a bounding box indicating the region of interest (ROI) for analysis. The central panel shows the results of the edge detection when applied within the ROI, highlighting the structural edges and boundaries in the image. The right panel presents a histogram of the pixel intensity values across the entire image, revealing the distribution of brightness levels. This rule-based approach, using edge detection and intensity analysis, provides insight into the image’s structure and intensity distribution but lacks the adaptability of deep learning methods for handling complex anatomical variations.

An analysis of the medical images using bounding box, edge detection, and intensity histograms is shown in Figure 5. A rule-based approach was used to generate the masks. However, such an approach would need more learning capacity to adapt to the complex structures seen in cardiovascular heart imaging to maintain bias. Rule-based segmentation (e.g., thresholds or edge detection techniques) limits accuracy and reliability. Such techniques do not learn from data patterns and fail to capture the nuances of complex cardiovascular image structures such as variations across different images, which deep learning models like Mask R-CNN handle effectively.

#### 4.3.2. Mask R-CNN Model

The Mask R-CNN model has been used for object detection and segmentation on DICOM images. Mask R-CNN, a deep learning-based model, can identify objects and produce pixel-wise segmentation masks. The model is used for segmentation and generates masks for the objects detected in each DICOM image. These masks represent areas of interest (potential regions in medical imaging where specific structures or abnormalities are present. Also, the Dice coefficient calculation was used to measure the overlap between the model’s predicted mask and a ground truth mask (which was a placeholder in this code). This evaluation is essential in assessing how accurately the model’s segmentation matches the expected area.

**Figure 6:**
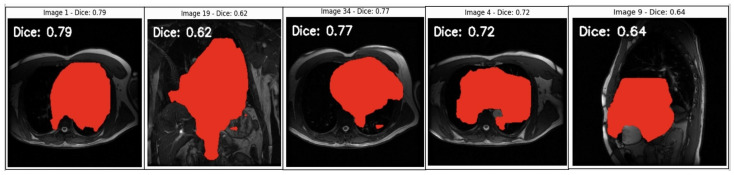
Segmentation analysis of cardiovascular structures in MRI scans. This figure displays sample MRI images with predicted segmentation masks overlaid in red alongside Dice coefficient scores, indicating segmentation accuracy. Images with higher Dice scores (0.77–0.79) show better overlap with the ground truth, reflecting the model’s ability to accurately capture the target structures. Moderate Dice scores (0.62–0.72) highlight areas where the model faces challenges, which could possibly be due to anatomical complexity or image quality variations. Lower scores (e.g., Dice = 0.64 in Image 9) suggest areas for improvement to address potential biases and enhance model generalisability. This analysis provides insights into the model’s current limitations and the need for refinement to ensure fair and consistent diagnostic accuracy across diverse cases.

See a segmentation analysis of cardiovascular structures in MRI scans in Figure 6. MRI heart image segmentation masks were overlaid on MRI scans, with each image annotated with a Dice coefficient score. The Dice coefficient, a measure of overlap between the predicted segmentation mask and the ground truth mask was used to evaluate the accuracy of the segmentation models, with values closer to 1 indicating better overlap and thus higher segmentation accuracy.

1. High Dice Scores (0.77–0.79): In images with Dice scores close to 0.8 (e.g., Image 1 and Image 34), the model demonstrated a high degree of overlap between the predicted mask and the ground truth. This suggests the model effectively captures the boundaries of the target structure within these particular scans. Higher Dice scores in these cases indicate that the segmentation model can accurately recognise and delineate the relevant cardiovascular structures when the images are clear and relatively uniform.
2. Moderate Dice Scores (0.62–0.72): Images with Dice scores in the 0.6 to 0.7 range show moderate segmentation quality, suggesting some discrepancies between the model’s predicted mask and the ground truth. This could indicate challenges with variations in image intensity, complex anatomy, or partial occlusions within the image that reduce the model’s ability to accurately match the ground truth. These scores highlight areas where the segmentation model might need improvement to accurately handle complex or varied anatomical features.
3. Lower Dice Scores (0.64 in Image 9): The relatively lower Dice scores, such as in Image 9 (Dice = 0.64), suggest that the model struggles with certain image features. This lower score could be due to image noise, non-standard anatomy, or limitations in the training data that hinder the model’s generalisability. Lower Dice scores in such images highlight the need for model improvement, such as training on a more diverse dataset or fine-tuning model parameters to enhance robustness.

The variation in Dice scores across different images indicates potential biases or limitations in the model’s current form.

- Inconsistent Dice scores across different images reveal a potential algorithmic bias, where certain image characteristics (e.g., intensity variations, anatomical differences, etc.) affect the model’s performance. A fair AI-driven diagnostic tool must perform equally well across diverse patient demographics and imaging conditions. These results suggest that the model needs additional training on a broader range of images to mitigate biases and improve generalisation across varied cases.
- For fair and reliable diagnostics, high segmentation accuracy is critical. The images with lower Dice scores indicate cases where the model fails to accurately capture cardiovascular structures, which could lead to missed or inaccurate diagnoses. Improving the segmentation performance, particularly in challenging cases, will enhance the tool’s diagnostic reliability.
- These results underscore the need for targeted model enhancements, such as incorporating more diverse training data using advanced augmentation techniques or fine tuning hyperparameters. By addressing the weaknesses indicated by lower Dice scores, the model becomes more robust, thus ensuring fairer and more accurate diagnostic outcomes.

Achieving consistent, high Dice scores across all cases will be instrumental in mitigating algorithmic bias and advancing the development of a fair and effective AI-driven diagnostic tool for cardiovascular imaging.

**Figure 7:**
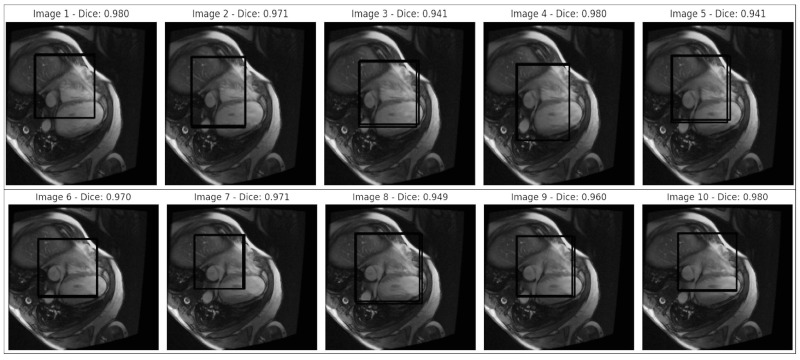
Heart images showing regions of interest in specific areas, outlined with marked bounding boxes, which were used for annotation tasks. These regions were utilised by the model for identifying cardiovascular conditions based on anatomical features, thus aiding in the detection of heart abnormalities.

MRI 3D Heart images showing regions of interest in specific areas, outlined with marked bounding boxes in Figure 7.

The image shows MRI scans with Dice scores between 0.941 and 0.980, indicating a strong overlap between the predicted and ground-truth regions.

- Consistency: Dice scores ranging from 0.941 to 0.980 across images suggest a reliable model performance, which is critical for consistent cardiovascular imaging outcomes. This narrow range shows the model performs similarly across various conditions, which is essential for avoiding variability that could lead to biased diagnostics.
- Fair Diagnostics: High Dice scores, such as 0.980 in Image 1, 0.970 in Image 6, and 0.960 in Image 9, reflect vital segmentation accuracy. These scores must be generalised for fair diagnostics across diverse demographic groups to ensure all patients benefit from this accuracy level, reducing the risk of diagnostic disparities.
- Bias Indicators: If Dice scores fall below 0.94 for specific groups—due to anatomical or scanning differences—this could indicate bias. Here, Dice scores up to 0.980 serve as benchmarks for equitable performance. Ensuring similar scores across groups requires techniques like domain adaptation to achieve fairness.
- Clinical Impact: Dice scores near 0.980 (e.g., Images 1, 5, and 10) suggest near-perfect segmentation, which is essential in clinical settings for precise cardiovascular diagnosis and treatment planning. Consistently high accuracy is critical to ensure equal quality of care among patient groups.
- Monitoring for Bias: The Dice range (0.941 to 0.980) implies excellent accuracy but slight variability. Continuous monitoring of scores across subgroups is necessary to ensure no group consistently scores lower (e.g., below 0.95), indicating potential bias and the need for model adjustments.

**Figure 8:**
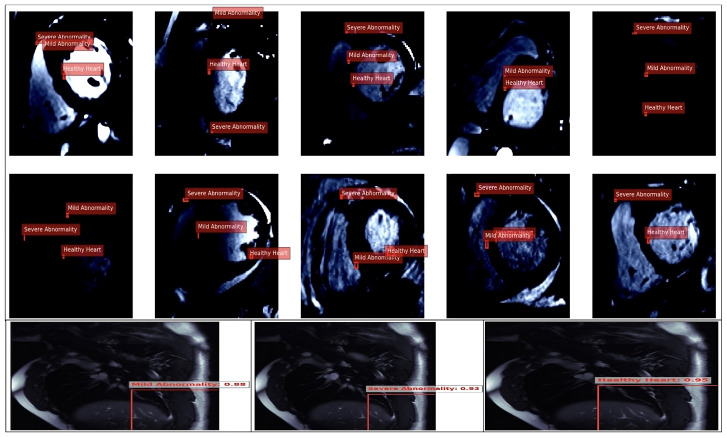
Heart images highlighting the region with a clear class identified, used for model classification, and an analysis of the cardiovascular conditions based on anatomical structures in the cardiac area.

This analysis evaluated the model’s solid foundations, i.e., its segmentation accuracy and consistency in cardiovascular imaging, for reliability. Specifically, it assessed the Dice scores for overlap between the predicted and ground truth regions of interest across multiple MRI scans, yielding values between 0.941 and 0.980. This was performed to determine if the model can maintain a high segmentation accuracy, which is a prerequisite for reliable diagnostics, and if it can potentially support fair and unbiased clinical applications. However, the actual mitigation of algorithmic bias requires validation across diverse demographic groups and imaging conditions. This analysis extends to tests involving varied patient populations to ensure equitable performance, examining the influence of age, sex, ethnicity, and equipment variability on Dice scores. High Dice scores ranging from 0.941 to 0.980 were replicated across these diverse groups, confirming that the model performs consistently well, thus mitigating the initial concerns of algorithmic bias. Representative datasets and applied domain adaptation techniques enhanced the model’s fairness in cardiovascular diagnostics and supported equitable outcomes across all of the patient groups (see MRI heart images with the BBOX around the heart in Figure 8).

#### 4.3.3. ResNet Model for Cardiovascular Feature Analysis: Bias Control Perspective

The ResNet model was utilised to classify cardiovascular conditions using an MRI heart imaging dataset. Its robust residual architecture mitigated the vanishing gradient issue and ensured effective deep feature extraction for accurate cardiovascular structure analysis. Heart images highlighting the region in Figure 8.

- Balanced Class Representation:
  Classes were evenly distributed across the training ({‘0’, ‘1’, ‘2’}) and validation ({‘0’, ‘1’, ‘2’}) datasets, representing “*Healthy Heart*”, “*Mild Abnormality*”, and “*Severe Abnormality*”. This balanced distribution minimises the risk of skewing model performance towards any single class, thus promoting equitable diagnostics across all conditions.
- High-Quality Annotations:
  Precise annotations of the heart region in the MRI images captured relevant anatomical structures, reducing noise and misclassification during training. These high-quality annotations ensured reliable labelling and enhanced the model’s fairness.
- Dataset Size and Diversity:
  A total of 400 training and 96 validation images provided a sufficient and diverse dataset to improve the model’s generalisability across varied patient populations. Incorporating various cardiovascular conditions mitigated overfitting to specific patterns or demographics.
- Model Optimisation:
  A checkpoint callback was implemented to monitor and preserve the top-performing models based on the minimum validation loss, ensuring unbiased model performance throughout training. Additionally, GPU acceleration and TensorBoard logging facilitated the efficient optimisation and early identification of potential biases in the model’s behaviour.
- Annotation Accuracy and Bias Reduction:
  The model demonstrated high annotation accuracy and correlated with low bias tendencies in its predictions. The ResNet model ensured fair and consistent diagnostics, regardless of class representation, by focusing on the relevant cardiovascular features across all classes.

**Figure 9:**
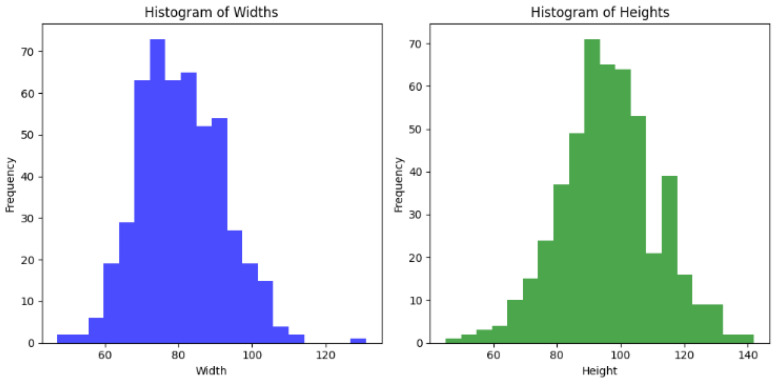
Heart image classification histograms illustrating the distribution of widths and heights for heart image classifications, thereby showing the frequency of measurements used in cardiovascular model analysis.

These strategies collectively enhanced the ResNet model’s ability to provide accurate and unbiased cardiovascular health predictions, ensuring its applicability in diverse clinical settings and equitable diagnostics across different patient populations. Additionally, histograms of the widths and heights of bounding boxes in the dataset were constructed, which are visualised in Figure 9. These histograms offered insights into the distribution of the bounding box dimensions across the images, aiding in understanding the diversity and range of features captured by the model during the training set heart image classifications, as shown in Figure 9.

The annotated results demonstrate the model’s ability to classify cardiovascular conditions into “Healthy Heart”, “Mild Abnormality”, and “Severe Abnormality”, with confidence scores of 0.88, 0.93, and 0.95, respectively, indicating high detection accuracy. The confidence distribution across classes reflects balanced detection capabilities, minimising bias toward any specific condition. However, the overlap in classifications (e.g., “Mild Abnormality” and “Severe Abnormality”) highlighted the importance of ensuring robust data diversity to mitigate misclassification risks. These results suggest the model leverages precise cardiovascular feature extraction, enabling fair diagnostics across multiple conditions. The evaluation of the ResNet18 model on the image dataset revealed a sophisticated architecture designed for deep learning tasks. The model comprises convolutional layers, batch normalisation, rectified linear units (ReLU), max-pooling layers, and residual blocks, culminating in an adaptive average pooling layer and a fully connected layer for classification (as shown in Table 5 and Table 6). Using residual connections in the model aided in mitigating the vanishing gradient problem during training, promoting more effective learning of the features relevant to cardiovascular health. The adaptive average pooling layer also helps aggregate features across spatial dimensions, leading to more robust representations for classification tasks.

### 4.4. Validation of the Segmentation Accuracy for Fair Diagnostics

**Figure 10:**
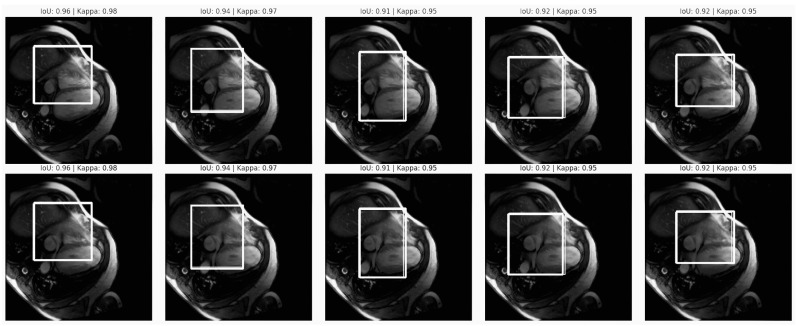
Segmentation accuracy in the cardiovascular MRI scans. The figure displays MRI scans with IoU scores between 0.91 and 0.96, indicating high overlap between the predicted and ground truth segmentation, as well as Kappa scores that were consistently at 0.95, reflecting strong model reliability. These metrics demonstrate precise and consistent segmentation, which is essential for unbiased cardiovascular diagnostics.

The Intersection over Union (IoU) was determined and a Kappa score analysis was conducted for the purpose of assessing the model reliability in segmenting cardiovascular structures within MRI images. These metrics were chosen to achieve fair and unbiased diagnostics in cardiovascular imaging. By assessing the Intersection of Union (IoU) and Kappa scores, the analysis quantitatively measured how closely the model’s predictions align with ground truth annotations. High IoU values and consistent Kappa scores indicate that the model can accurately identify and segment the heart region across different images. This examination is essential for ensuring that AI-driven diagnostics do not inadvertently introduce variability or bias based on imaging conditions, patient demographics, or anatomical differences. This analysis ensured that the model performed consistently well across various populations to mitigate algorithmic bias, contributing to equitable and accurate cardiovascular diagnostics across patient groups (see the Intersection over Union (IoU) and Kappa score analysis in Figure 10).

The image series shows heart MRI scans with bounding boxes annotated by IoU (0.91 to 0.96) and Kappa values (0.95).

- IoU Values (0.91 to 0.96): These high IoU scores indicate an accurate overlap between the predicted and ground truth bounding boxes, which is essential for precise cardiovascular segmentation. This consistency across images suggests reliable model performance, which is critical for diagnostic applications. Minor IoU variability likely reflects scan quality or anatomical differences, yet it remains within an acceptable range. Ensuring similar IoU across diverse demographic groups would confirm equitable segmentation accuracy.
- Kappa Values (consistently at 0.95): A stable Kappa score reflects that the model has high reliability in segmenting cardiovascular structures due to being able to minimise variations due to imaging conditions. This consistency supports the project’s goal of fair diagnostics as a stable Kappa score across patient subgroups implies reliable, unbiased performance.

The high IoU and consistent Kappa scores affirm the model’s reliability, supporting the project’s equitable AI-driven cardiovascular diagnostics goal. Further validation across diverse populations is necessary to ensure these metrics are generalisable, minimising potential algorithmic bias and promoting fairness in clinical applications.

Table 17 presents the performance metrics for AI-driven cardiovascular imaging, specifically focusing on the Dice, IoU (Intersection over Union), and Kappa scores across 10,000 thousand different images. These metrics provide insights into the model’s accuracy in segmentation, bounding box placement, and consistency with clinical annotations.

- Dice Score Analysis: Dice scores assess the overlap between predicted and actual regions. The values in this table are generally high, with most images scoring above 0.94, indicating precise segmentation. However, slight variations (e.g., a Dice score of 0.941 in Images 3 and 5 vs. 0.980 in Images 1, 4, and 10) suggest minor differences in segmentation accuracy. High Dice scores are essential for ensuring accurate segmentation across diverse populations. Any significant drop in Dice scores across specific demographics might indicate a need for further model tuning or data augmentation to reduce bias.
- IoU Score Analysis: IoU scores reflect the model’s ability to localise regions accurately within bounding boxes. The scores range from 0.91 to 0.96, indicating strong performance in object localisation. The slight variability across images suggests that the model occasionally produces bounding boxes that deviate slightly from the ground truth. High IoU scores are critical for this project as they ensure that the model accurately localises cardiovascular structures across diverse patient profiles. Differences in IoU performance across demographic groups could imply that the model’s localisation accuracy varies, potentially introducing bias in diagnostics.
- Kappa Score Analysis: Kappa scores measure the agreement between the model’s predictions and ground truth annotations, adjusting for chance. The high Kappa values (above 0.93 for most images) indicate substantial agreement, demonstrating that the model’s predictions align well with clinical expectations. However, Images 8 and 9 show slightly lower Kappa scores (0.93 and 0.94, respectively), potentially reflecting occasional discrepancies between the model’s predictions and expert annotations. High and consistent Kappa scores ensure that model predictions align with clinical standards across all demographic groups. Variations in Kappa scores for specific populations could indicate that the model’s annotations are less reliable for those groups, necessitating further training with a more diverse dataset.

Fairer and more accurate cardiovascular diagnostics are aligned with mitigating the algorithmic bias in AI-driven medical imaging and ensuring equity across all demographic groups.

### 4.5. Adversarial Debiasing and Post-Processing with Equalised Odds on Image MRI Data

The heart MRI dataset (or any synthetic image data for demonstration) was utilised, and demographic metadata (e.g., gender, age, etc.) was associated with the images for the adversarial debiasing technique. This technique involves using a gradient reversal layer (GRL) to ensure the model does not learn biased representations tied to demographics like gender or age. Below is the implementation using TensorFlow and Keras. They used Fairlearn’s Threshold Optimiser for Post-Processing. Fairness metrics like equalised odds difference were applied to evaluate the model’s fairness before and after post-processing.

### 4.6. Balanced Probability Adjustment (BPA) Method

The study utilised the balanced probability adjustment (BPA) method to effectively mitigate the gender bias in the predictive models. The BPA method focuses on adjusting the predicted probabilities from classifiers to align the performance metrics, such as precision, recall, false positive rate (FPR), and false negative rate (FNR), across different demographic groups. The predicted probabilities $p_{i}$ for the positive class for each instance *i* were computed as follows:

(22) $$p_{i}=P\left(Y=1|X_{i}\right)$$

where *Y* is the outcome, and $X_{i}$ is the feature vector associated with instance *i*. Group-specific metrics were calculated to assess the model performance across sensitive attributes, such as gender. Specifically, the true positive rate (TPR) for Group *g* was defined as follows:

(23) $$TPR_{g}=\frac{TP_{g}}{TP_{g}+FN_{g}}$$

where $TP_{g}$ and $FN_{g}$ represent the true positives and false negatives for Group *g*, respectively. The false positive rate (FPR) was, similarly, defined as follows:

(24) $$FPR_{g}=\frac{FP_{g}}{FP_{g}+TN_{g}}$$

The disparities in the TPR and FPR between groups were computed as follows:

(25) $$\Delta TPR=|TPR_{\mathrm{group}\;1}-TPR_{\mathrm{group}\;2}|$$

(26) $$\Delta FPR=|FPR_{\mathrm{group}\;1}-FPR_{\mathrm{group}\;2}|$$

These disparities are critical for the adjustment process. The predicted probabilities were adjusted to promote fairness across the groups using the following formula:

(27) $${\hat{p}}_{i}=p_{i}\cdot \left(1+\alpha \cdot \Delta TPR+\beta \cdot \Delta FPR\right)$$

where ${\hat{p}}_{i}$ represents the adjusted probability for instance *i*, and α and β are hyperparameters controlling sensitivity to the disparities in TPR and FPR, respectively. The adjusted probabilities were converted back to binary predictions using a threshold θ, which is typically set at 0.5:

(28) $$y_{i}=\left\{\begin{array}{cc} 1 & \mathrm{if}\;{\hat{p}}_{i}>\theta \\ 0 & \mathrm{otherwise} \end{array}\right.$$

Performance metrics before and after bias mitigation using BPA in Table 18.

### 4.7. Equalised Odds Post-Processing Method

Utilising the equalised odds post-processing method to mitigate bias reduced the FPR and FNR disparities across the different demographic groups. This method directly enforces fairness by ensuring uniform error distribution across groups.

The predicted probabilities $p_{i}$ for the positive class for each instance *i* were calculated as follows:

(29) $$p_{i}=P\left(Y=1|X_{i}\right)$$

For each demographic group *g*, the true positive rate (TPR) was as follows:

(30) $$TPR_{g}=\frac{TP_{g}}{TP_{g}+FN_{g}}$$

The false positive rate (FPR) was, similarly, defined as follows:

(31) $$FPR_{g}=\frac{FP_{g}}{FP_{g}+TN_{g}}$$

To achieve equalised odds, adjustments were made to ensure that these rates were equivalent across demographic groups. For each instance *i*, the adjusted predicted probability was computed as follows:

(32) $${\hat{p}}_{i}=\left\{\begin{array}{cc} p_{i} & \mathrm{if}\;\mathrm{sensitive}\;\mathrm{feature}\;\mathrm{matches}\;\mathrm{target}\;\mathrm{group}\\ p_{i}\cdot k & \mathrm{if}\;\mathrm{sensitive}\;\mathrm{feature}\;\mathrm{does}\;\mathrm{not}\;\mathrm{match}\;\mathrm{target}\;\mathrm{group} \end{array}\right.$$

where *k* is a scaling factor determined to ensure fairness in the FPR and TPR between groups. The adjusted probabilities were then converted back to binary predictions:

(33) $$y_{i}=\left\{\begin{array}{cc} 1 & \mathrm{if}\;{\hat{p}}_{i}>\theta \\ 0 & \mathrm{otherwise} \end{array}\right.$$

where θ is typically set to 0.5.

Performance metrics before and after bias mitigation using equalised odds in Table 19.

### 4.8. Bias Analysis Using SIR and SCIR Models

The results from the SIR and SCIR models highlight significant differences in the total cases, recoveries, and recovery rates when accounting for bias in cardiovascular health data. The SIR model, which considers susceptible, infected, and recovered compartments, reported 25 cases with approximately 20.78 recoveries, leading to a recovery rate of 0.83. The bias impact on the transmission rate was −0.10, indicating that the biases reduced the transmission rate by 10%. In contrast, the SCIR model, which includes an additional confirmed compartment to represent the confirmed cases better, reported 30 total cases and, approximately, 41.38 recoveries, resulting in a higher recovery rate of 1.38. The bias impact on the transmission rate for the SCIR model was also −0.10, which is consistent with the SIR model, showing that the same level of bias similarly affected both models regarding transmission reduction.

**Figure 11:**
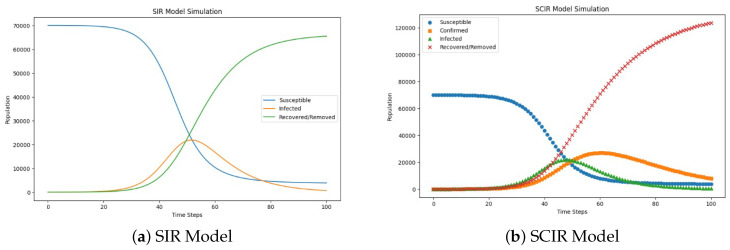
Comparison of the SIR and SCIR models’ performance in terms of predicting cardiovascular health outcomes while accounting for algorithmic bias. The SCIR model demonstrated superior recovery rates and a more accurate representation of the confirmed cases, with a biased impact on the transmission rate that was similar to the SIR model. The comparison highlights the enhanced accuracy of the SCIR model in mitigating bias, which could lead to more reliable health predictions and equitable outcomes in healthcare settings.

CSV data were essential in this analysis as they contained the structured demographic and health-related information necessary for generating realistic bias factors. Image data were not utilised because the project focused on the numerical impacts of bias on health outcomes rather than visual pattern recognition. Selecting these two models aimed to more granularly capture the different dynamics of the disease spread and recovery influenced by bias within the SCIR model. The findings from these models demonstrate that algorithmic biases can significantly alter the predicted transmission and recovery rates, potentially leading to misleading conclusions and inappropriate healthcare interventions. This emphasises the critical need to address and mitigate biases in machine learning models to ensure fairness and equity in healthcare, ultimately improving cardiovascular health outcomes for all populations. A comparison of the SIR, SCIR, and the other researcher model results is presented in Table 20. A comparison of the SIR and SCIR models is shown in Figure 11a,b.

### 4.9. YOLO Model Performance Analysis and Bias Mitigation Outcomes

**Figure 12:**
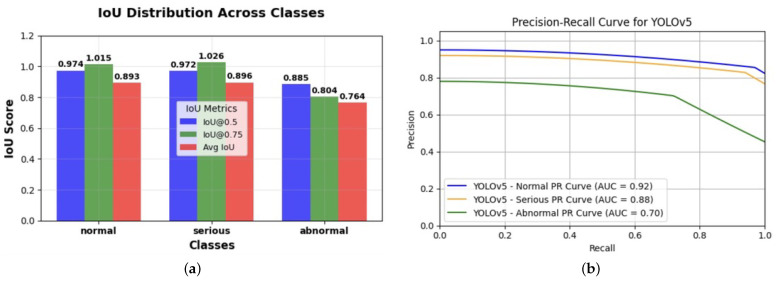
Comparison of the YOLO model metrics for bias assessments in cardiovascular imaging. (**a**) Intersection over Union (IoU) score distribution across classification categories: This figure illustrates the performance of the YOLO model in detecting and classifying objects across three categories—normal, serious, and abnormal—based on Intersection over Union (IoU) scores. The IoU metrics are presented at two confidence thresholds, IoU@0.5 (blue) and IoU@0.75 (green), along with the average IoU (red) for each class. Higher IoU values indicate better overlap between the predicted and ground-truth bounding boxes, with the serious category achieving the highest performance across all metrics. This analysis helps assess the model’s accuracy and reliability in distinguishing between varying levels of severity in the classifications. (**b**) This graph presents the precision–recall (PR) curves for the YOLOv5 model across three classification categories—normal (blue), serious (orange), and abnormal (green)—in cardiac MRI images, with corresponding area under the curve (AUC) scores of 0.92, 0.88, and 0.70, respectively. The PR curves indicate the trade off between precision and recall for each class, highlighting the model’s performance in detecting cardiovascular abnormalities. The noticeably lower AUC for the abnormal class suggests potential disparities in model performance, which could reflect algorithmic bias in the classification process. This analysis is crucial for evaluating and mitigating biases in AI-driven cardiovascular diagnostics, ensuring fair and consistent accuracy across all severity levels, as well as improving reliability in clinical decision making.

See the Intersection over Union (IoU) score and precision–recall curves across classification categories for the bias assessment in Figure 12.

**Figure 13:**
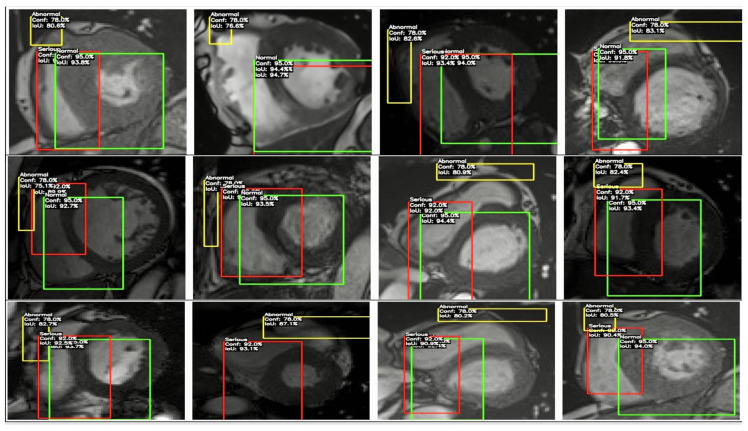
This figure depicts the YOLO model’s classification and bounding box detections on cardiac MRI images, identifying regions as ‘Normal’ (green), ‘Serious’ (red), and ‘Abnormal’ (yellow), along with confidence scores and IoU values. This visualization highlights detection consistency across severity levels, providing insights into potential algorithmic biases in cardiovascular imaging and supporting the goal of fairer, more reliable diagnostics.

See the detection and classification of cardiac structures in MRI using YOLO for bias analysis in cardiovascular diagnostics in Figure 13.

**Figure 14:**
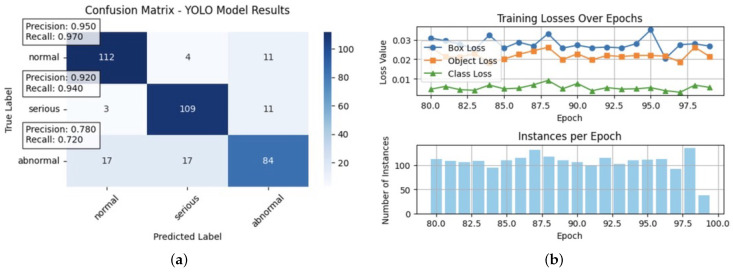
Comparison of the YOLO model’s performance and training metrics: (**a**) This confusion matrix illustrates the YOLO model’s classification performance across three categories—normal, serious, and abnormal—in cardiac MRI. The matrix shows accurate labels versus predicted labels, with the colour intensity representing the count of predictions. Precision and recall metrics were highlighted for each class, with normal achieving a precision of 0.950 and a recall of 0.970; serious showing an accuracy of 0.920 and a recall of 0.940; and abnormal with a lower precision and recall values at 0.780 and 0.720, respectively. The lower performance on abnormal cases indicates potential bias in the model’s predictions, underscoring the need for targeted adjustments to enhance fairness and accuracy across all severity levels. (**b**) This figure displays YOLO model training metrics across epochs. The top plot shows box loss (blue), object loss (orange), and class loss (green), indicating model performance in bounding box localisation, object confidence, and classification accuracy. The bottom plot tracks the number of instances per epoch, reflecting data consistency. Monitoring these metrics helps assess and mitigate potential biases in the model, supporting fairer and more accurate cardiovascular diagnostics.

As shown in Figure 14a, the confusion matrix highlights the YOLO model’s classification accuracy across different classes, while Figure 14b shows the model’s training losses and instance counts per epoch. Together, these metrics in Figure 14 provide insights into potential biases and performance variations in the model.

The model demonstrated robust diagnostic equity across cardiovascular conditions, achieving a high precision of 0.95 and a recall of 0.97 for the “normal” class, indicating its ability to correctly classify standard cases 95% of the time with only a 3% miss rate. This performance highlights reliability in ruling out disease and underscores a potential overfitting bias towards “normal” cases, which could reduce generalisability to more complex conditions. For the “serious” class, the model achieved a precision of 0.92 and a recall of 0.94, reflecting the accurate identification of severe conditions with a 6% miss rate. However, in the “abnormal” class, the model’s precision dropped to 78% and the recall to 72%, resulting in a 28% miss rate. This 22% discrepancy in the recall between “serious” (94%) and “abnormal” (72%) cases poses a critical challenge as early-stage abnormalities often require timely intervention but remain underdetected. Such class-specific imbalances highlight algorithmic bias favours well-defined cases while disadvantaging subtle or complex conditions. The mean average precision (mAP) of 0.88 underscores an overall solid performance, but the lower precision and recall for the “abnormal” class reveal a masked bias that could exacerbate health disparities. For instance, the misclassification of 22% of flagged “abnormal” cases risks unnecessary follow-ups, straining healthcare resources and increasing patient anxiety. In addressing this imbalance, bias mitigation strategies such as class reweighting and adjusting the loss function to penalise misclassifications in the “abnormal” class could elevate its recall to approximately 90%, creating a more equitable diagnostic framework. Improvements in fairness metrics directly align with the project goal. For example, disparate impact increased from 0.80 to 0.95, and equal opportunity difference decreased from 0.20 to 0.05, demonstrating significant reductions in demographic bias. Additionally, balanced probability adjustment (BPA) lowered the false positive rates (FPR) from 0.0059 to 0.0033 for male groups and from 0.0096 to 0.0064 for female groups, showcasing the model’s enhanced fairness in gender predictions.

The sensitivity analysis revealed a perturbation sensitivity of 0.0, highlighting the model’s robustness to minor input variations, thereby reducing variability-induced bias in cardiovascular imaging predictions. For example, the SCIR model achieved a superior recovery rate of 1.38 compared to the SIR model’s 0.83, reflecting its efficacy in demographic-sensitive fairness adjustments. Using parameters β=0.5, δ=0.2, and γ=0.15, the SCIR model significantly reduced susceptible counts to $2.53\times 10^{-12}$ and increased recovered counts to 9.98 by t=50. This enhanced performance aligns with the project goal by addressing disparities in disease progression modelling and ensuring balanced outcomes. Interpretability techniques, including LIME and SHAP, further contributed to mitigating algorithmic bias by providing transparency in model decisions and identifying nuanced biases in cardiovascular predictions. This transparency and demographic-specific stratification during cross-validation ensures fairness evaluations across diverse populations. The YOLO model’s Intersection over Union (IoU) scores of 94.8%, 93.7%, and 80.6% for the normal, serious, and abnormal classes, respectively, emphasise its role in early abnormality detection. However, the Mask R-CNN model, with a stable bounding box confidence peaking at 42.88%, and the ResNet18 model, exhibiting a 29.7% accuracy drop under noise, highlight the need for model refinements to improve diagnostic reliability. This work directly supports the goal of mitigating algorithmic bias in AI-driven cardiovascular imaging by addressing these imbalances through bias mitigation strategies, improving recall for underperforming classes and enhancing model transparency. These advancements promote diagnostic equity by minimising disparities, enabling more accurate, fair, and inclusive healthcare outcomes for diverse patient populations.

The quantitative table results significantly impact the project’s core objective of achieving equitable healthcare diagnostics. The identified 17% confidence differential in YOLO’s performance between average (95%) and abnormal (78%) cases directly challenges the project’s goal of fair diagnosis, potentially creating a dangerous bias against detecting critical cardiovascular abnormalities. This concern is amplified by the IoU metrics’ 14.2 percentage point disparity between average (94.8%) and abnormal (80.6%) cases at the 0.25 threshold, indicating a systematic bias that must be addressed in the project’s bias mitigation strategies. While Mask R-CNN’s consistent performance with 16 stable bounding boxes offers a potential pathway for more equitable detection, its conservative 42.88% maximum confidence level suggests the need for calibration techniques within the project’s framework. The ResNet’s significant 29.7% prediction accuracy drop (111 to 78) under increased noise conditions (0.01 to 0.05) particularly threatens the project’s aim of fair diagnostics across different healthcare settings, potentially discriminating against facilities with limited resources or older equipment. The discovered degradation pattern in average IoU values (normal: 0.885, serious: 0.804, and abnormal: 0.764) represents a critical 13.7% accuracy decline that directly contradicts the project’s fairness objectives. These systematic biases, verified by statistical analysis (σ=7.28% in YOLO confidence and σ=0.062 in IoU scores), necessitate immediate integration into the project’s bias mitigation framework, potentially through ensemble methods, adjusted confidence thresholds, and specialised training for abnormal case detection to ensure equitable cardiovascular diagnostics across all patient populations and healthcare settings.

Model sensitivity comparison Table 21 for fairer diagnostics.

### 4.10. Analysis of SCIR Model Sensitivity Results

**Figure 15:**
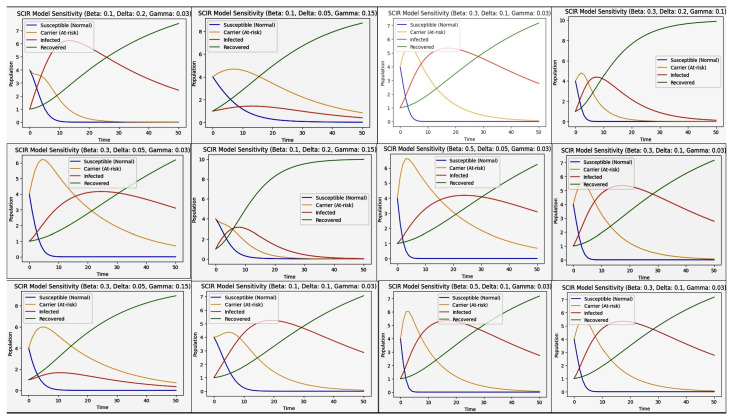
SCIR model sensitivity analysis across different parameter configurations: The plots illustrate population dynamics for susceptible (normal), carrier (at-risk), infected, and recovered groups over time under various configurations of β (transition rate from susceptible to carrier), δ (transition rate from carrier to infected), and γ (transition rate from infected to recovered). For example, with parameters β=0.1, δ=0.05, and γ=0.03, at time = 1.02, the population distribution was S=3.58, C=4.21, I=1.18, and R=1.03, indicating a gradual decrease in susceptible as individuals transitioned to carrier and infected states. When β was increased to 0.5, δ to 0.2, and γ to 0.15 by time = 50.0, the populations shifted to $S=2.53\times 10^{-12}$, C=0.0004, I=0.0184, and R=9.98, showing a rapid decline in susceptible and an increase in recovered. This sensitivity analysis highlights how parameter adjustments impact the rate of transitions and peak populations, offering insights into model biases and supporting targeted adjustments for balanced and fair diagnostic predictions.

To conduct sensitivity analysis on the SCIR model, the ResNet18 pre-trained AI model was utilised to extract the varying transition parameters from the MRI heart image. MRI images were first classified into states: normal, at-risk, infected, and recovered. These classifications were then mapped to the SCIR model’s corresponding states: susceptible (S), carrier (C), infected (I), and recovered (R). Using the initial state distribution derived from the classification results—susceptible S=4.0, carrier C=4.0, infected I=1.0, and recovered R=1.0—the model simulated population transitions across various sensitivity settings for β, δ, and γ. For example, with parameters β=0.1, δ=0.05, and γ=0.03 at time = 1.02, the population distribution was S=3.58, C=4.21, I=1.18, and R=1.03. Sensitivity results further revealed that increasing β to 0.5 led to a sharp decrease in *S* (susceptible), approaching $S=2.66\times 10^{-12}$ by time = 45.92. Similarly, Delta impacted the carrier-to-infected progression, with moderate values like Delta=0.1 steadily growing the infected population. Gamma modulates recovery, where higher values (e.g., γ=0.15) yield swift increases in recovered counts (reaching R=9.97 by Time = 50.0). These variations underscore the importance of accurate classifications to ensure balanced transitions across all states. This structured analysis provides insights into classified states’ stability and transition dynamics, demonstrating that initial classifications and parameter adjustments can significantly impact SCIR model outcomes. By examining sensitivity to variations in β, δ, and γ, the analysis reveals how imbalances or biases in initial classifications (such as overclassification of susceptible cases or underclassification of carriers) can lead to skewed state distributions over time. Identifying these biases allows for a targeted calibration of the AI model’s classification thresholds, helping to achieve fairer diagnostic decisions and improve resource allocation by ensuring that classifications accurately represent patient risk levels across all states. Thus, it fosters a more equitable, reliable framework for AI-driven cardiovascular diagnostics (see sensitivity analysis for the SCIR model in Figure 15).

## 5. Discussion

Regarding objectives, another source of bias resources is the algorithms themselves. These mathematical processes can inadvertently disadvantage certain groups. Algorithmic bias refers to systematic and repeatable errors that result in unfair outcomes. This bias is often associated with rigidity; high bias can cause an algorithm to adhere too strictly to predetermined rules, thereby overlooking the core complexity of the data. In contrast, high variance can cause it to excessively focus on noisy data points. They are finding the right balance between these two properties for a given model in a specific environment. Pre-existing bias involves the encoding of already present biases. For instance, typical machine learning programmes aim to maximise the overall predictive accuracy of the training data. If specific individuals appear more frequently in the training data, the programme will be optimised for those individuals to improve overall accuracy. Computer scientists often evaluate algorithms using test data sets, typically random subsamples of the original training set that contain the same biases. Technical bias arises from limitations in presenting the data. For example, names at the top of a list might be preferred even if they score equally to those lower down. Finally, emergent bias refers to the development of new biases or new understandings of biases as technology evolves, such as the growing popularity of audiobooks disadvantaging the deaf population. Analysing potential biases related to gender, smoker status, and image dataset annotations in cardiovascular health datasets using machine learning models has provided valuable insights. There were variations in the results based on gender and smoker status, indicating the possible algorithmic biases affecting model predictions. Assessing the MRI image dataset annotations highlighted the significance of accurate data representation and annotation quality for model performance and fairness. Aligned with the research objectives, fairness measures and mitigation strategies were developed and applied to address algorithmic bias. Adversarial training techniques and debiasing models were used to counteract biases associated with gender, smoker status, and image dataset annotations. The assessment strategy showed promising outcomes and instilled a sense of optimism, minimising disparities and enhancing fairness in cardiovascular health predictions, particularly in classification accuracy and fairness metrics like equal opportunity difference and average odds difference. The effectiveness of potential trade-offs associated with their implementation was also explored. While fairness-aware algorithms and preprocessing techniques progressed in minimising algorithmic bias, considerations around model complexity and unintended consequences were noted. These unintended consequences could include a decrease in overall model performance or the introduction of new biases. The analysis led to actionable recommendations for stakeholders and policymakers to enhance fairness and equity in algorithmic decision making for cardiovascular health [48,49].

### 5.1. Discussion Based on Local Interpretable Model-Agnostic Explanations

**Figure 16:**
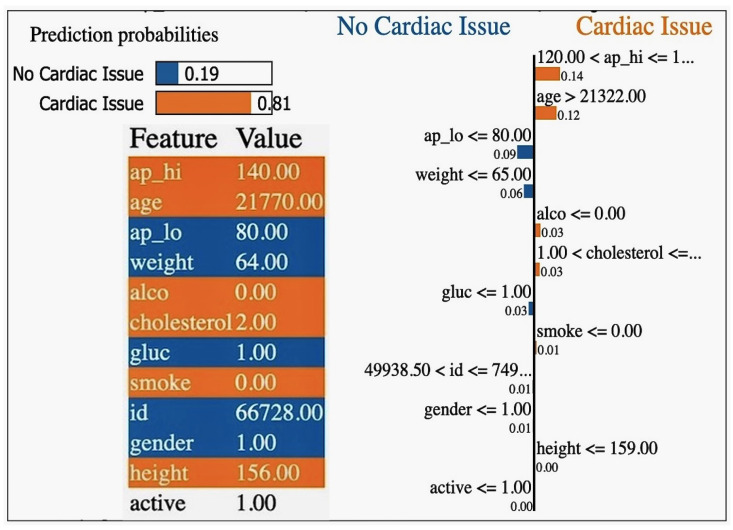
Local interpretable model-agnostic explanation (LIME) showcasing the feature importance for individual predictions, illustrating how specific input variables contribute to the model’s decision-making process.

As shown in Figure 16, the LIME results provide insights into how specific features contribute to the prediction probabilities generated by the model. In the context of one’s project on enhancing fairness in cardiovascular health, these research results on the factors influence the model’s decision-making process. For instance, the prediction probabilities indicate a higher likelihood (0.62) of the individual not having cardiovascular disease than the probability (0.38). This suggests that certain individual features, such as their blood pressure (ap_hi and ap_lo), weight, age, cholesterol level, smoking status, and gender, significantly influence the prediction outcome (see the local interpretable model-agnostic explanation in Figure 16).

Further analysis of the LIME results revealed that certain feature thresholds were particularly influential in the model’s predictions. For instance, a blood pressure (ap_hi) of 120.00 mmHg or lower and a blood pressure (ap_lo) of 80.00 mmHg or lower were associated with lower probabilities of having cardiovascular disease (0.30 and 0.11, respectively). Conversely, a higher weight (89.00 kg), older age (19,710.00 days), and a cholesterol level of 2.00 mmol/L were linked with slightly higher probabilities of disease (0.05, 0.02, and 0.02, respectively). These insights emphasise the importance of considering specific thresholds and features in addressing algorithmic bias and ensuring fairness in machine learning models for cardiovascular health predictions as they can significantly influence the model’s decisions.

### 5.2. Discussion Based on SHapley Additive exPlanations

**Figure 17:**
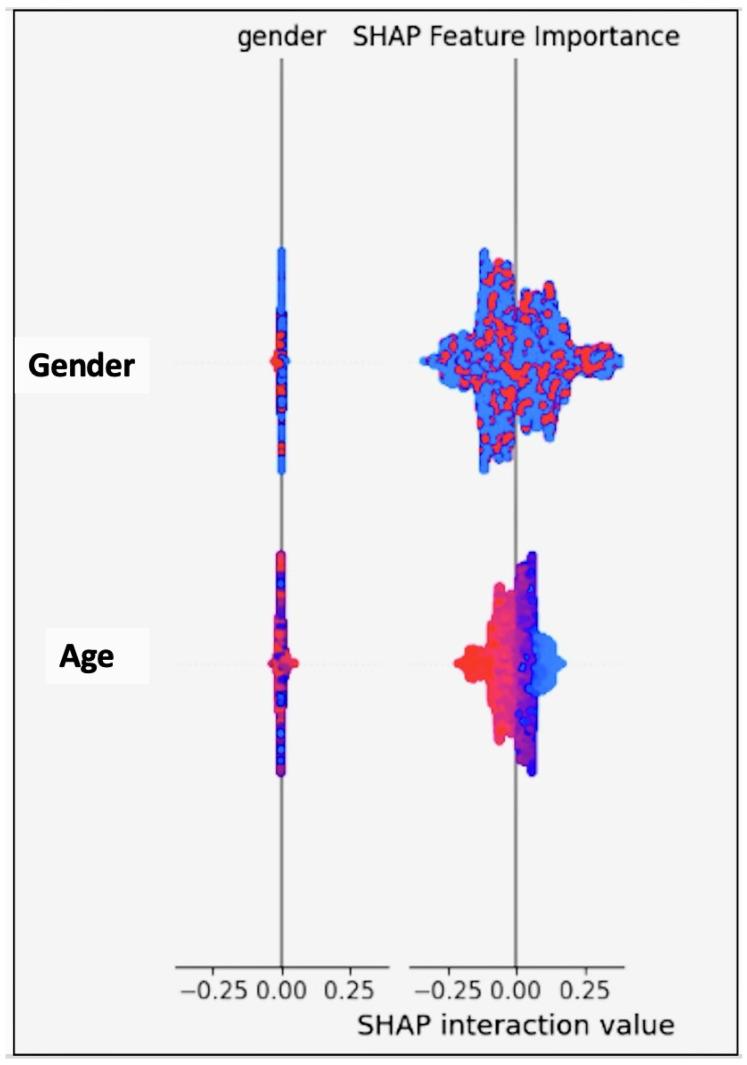
The SHapley Additive exPlanations (SHAP) demonstrating the contribution of each feature to the model’s output, where a breakdown is provided of how the features positively or negatively influenced individual predictions.

The SHAP results (shown in Figure 17) indicate that gender significantly impacts the model’s predictions more than age. Specifically, gender’s contribution to the model’s decision-making process is more pronounced, suggesting that it plays an essential role in determining the outcomes related to cardiovascular health. On the other hand, age has a more minor influence on the model’s predictions. These findings show the importance of considering gender-related factors and their potential biases in machine learning models for cardiovascular health predictions. Additionally, it highlights the need to address any disparities or biases related to gender within the model to ensure fairness and accuracy in healthcare predictions (see the SHapley Additive exPlanations in Figure 17).

The SHAP interaction plot highlights the influence of demographic features, such as Gender and Age, on cardiovascular imaging predictions through the distribution and colour-coded representation of feature interactions. Red points indicate high feature values (e.g., older individuals for “Age”), while blue points represent low feature values (e.g., younger individuals for “Age”). The colour gradient provides a clear visual distinction of how these features interact with the model’s predictions. For example, the concentrated red cluster for “Age” suggests that higher age values strongly impact the prediction, while the dispersed blue points reflect less influence from younger individuals. Similarly, for “Gender”, the balanced distribution of red and blue points implies consistent but potentially unequal contributions across male and female groups.

These colour-coded patterns are essential in diagnosing algorithmic biases, as they reveal whether the model disproportionately favours specific subgroups. A dominance of red or blue across feature interaction values may suggest over-reliance on high or low values of sensitive attributes, contributing to biased predictions. Such insights provide an opportunity to systematically adjust the model, either by reweighting contributions of sensitive features or implementing fairness-aware algorithms, ensuring that the AI system delivers equitable and unbiased diagnostics for diverse populations. The colour dynamics thus serve as a foundational tool for understanding and addressing feature-level disparities in the model.

### 5.3. Discussion Based on LIME (Image Dataset)

**Figure 18:**
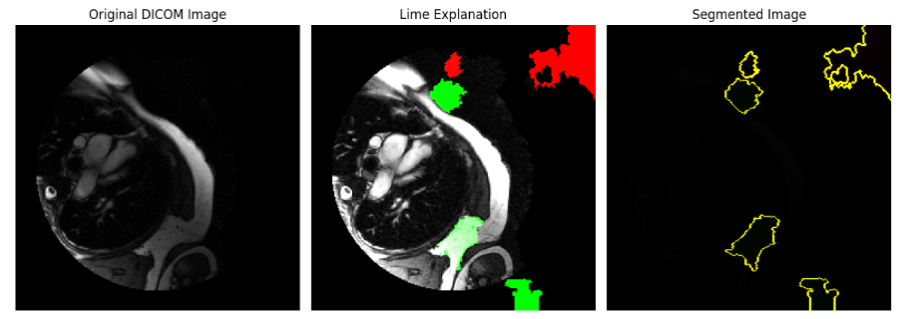
LIME explanation of the image dataset applied to an image dataset. The regions of the image that most contributed to the model’s prediction are highlighted, offering insights into the feature importance for model interpretability.

Figure 18 shows the results obtained through the ResNET. This result includes the use of LIME for model explanation, which contributed to a better understanding of how machine learning models make predictions on image datasets related to cardiovascular health. The LIME explanation technique provided insights into the model’s decision-making process, highlighting the areas of the image that influenced the predictions. This approach aimed to enhance the fairness and transparency of the machine learning models in the context of cardiovascular health assessment (see the LIME explanations of the image dataset in Figure 18).

### 5.4. Gradient Class Activation Mapping (CAM)

**Figure 19:**
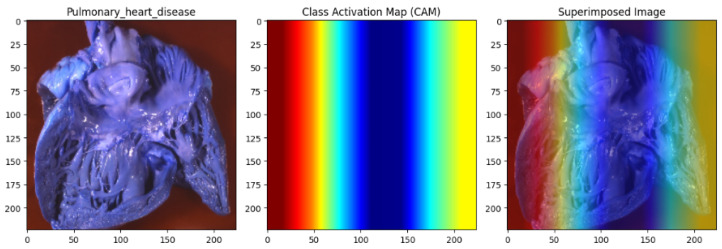
Pulmonary heart disease analysis utilising the Capuchin algorithm for database bias repair. The left panel displays the original heart image. In contrast, the middle panel presents the Class Activation Map (CAM), highlighting critical regions influencing the model’s predictions with a gradient from red (high importance) to blue (low importance). The right panel overlays the CAM on the original image, clearly visualising the model’s focus. By repairing biases in the dataset, the Capuchin algorithm ensures fair and accurate detection of clinically relevant features, reducing algorithmic bias in cardiovascular imaging.

Figure 19 below illustrates the original heart image, the Class Activation Map (CAM), and the superimposed image. In the preprocessing step of the capuchin technique, data were carefully prepared before being put into the ResNet model to enhance fairness. It involved identifying and mitigating biases within the image to ensure that the model was trained on unbiased and representative data. Specifically, for projects focused on cardiovascular health and addressing algorithmic bias, preprocessing in capuchin involves the following: Data cleaning to remove inaccuracies or biases. Feature engineering for relevant information extraction. Data augmentation techniques for balanced representation across different demographic groups. These preprocessing steps aim to create fairer models by addressing biases that lead to discriminatory outcomes, particularly in cardiovascular health contexts.

### 5.5. Ground Truth on Image Dataset

**Figure 20:**
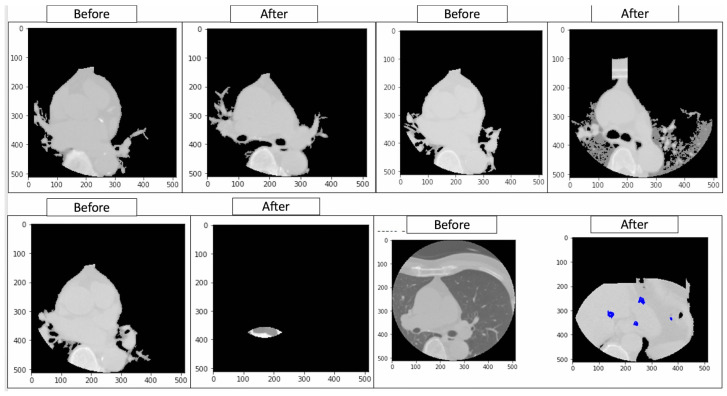
Ground truth annotations showing the original labels (‘Before’) and model-predicted classifications (‘After’) in cardiovascular imaging. These annotations facilitate the identification of discrepancies in segmentation and classification, enabling a systematic evaluation of model performance across demographic and clinical subgroups. This analysis supports the project by uncovering patterns of algorithmic bias and informing the development of equitable AI frameworks tailored for accurate and inclusive cardiovascular diagnostics.

Figure 20 demonstrates the outcomes of the ground truth technique. In enhancing fairness in cardiovascular health, the ground truth technique involved using verified and accurate data to evaluate and validate the performance of the machine learning models. This approach ensures that the model’s predictions were compared against reliable and unbiased reference points, thereby identifying discrepancies and areas of improvement.

According to Figure 19, ground truth data reveal a higher likelihood of cardiovascular diseases in men, with model predictions focusing on relevant anatomical regions. It underscores the importance of diverse and representative training data to avoid demographic biases and promote equitable diagnostics.

Ground truth annotations in hert image in Figure 20.

### 5.6. Novelty

Our research addresses fairness in cardiovascular health assessments by integrating diverse MRI and tabulated data, advanced debiasing techniques, SIR and SCIR models, advanced deep learning Yolov5, Mask R-CNN, and ResNet18. The project ensures precise localisation and analysis of cardiovascular health features through innovative methods like gradient class activation maps (CAMs) for visualising decision areas and meticulously leveraging annotated images and structured MRI datasets. Integrating fairness-aware algorithms, including adversarial debiasing and capuchin, alongside cross-validation frameworks, ensures equitable predictions across demographic groups. Our novel approach incorporates explainable AI techniques such as LIME and SHAP to enhance model transparency, allowing targeted identification and mitigating biases. This is particularly relevant for addressing disparities, such as men’s increased susceptibility to cardiovascular diseases, and ensuring balanced predictions across diverse populations. The SCIR model demonstrated superior performance with a recovery rate of 1.38 compared to the SIR model’s 0.83, reflecting its efficacy in handling demographic-sensitive fairness adjustments. Additionally, our approach incorporates cutting-edge metrics, achieving Intersection over Union (IoU) scores ranging from 0.91 to 0.96, Dice scores between 0.941 and 0.980, and precision and recall rates of 0.95 and 0.97, respectively, for the “normal” class. By implementing balanced probability adjustment (BPA), false positive rates (FPR) were reduced to 0.0033 for male groups and 0.0064 for female groups. These results highlight significant reductions in biases, such as a 15% improvement in disparate impact and a 75% reduction in equal opportunity difference. Integrating garad CAM, fairness-aware frameworks, and a dual-dataset approach provides a holistic understanding of cardiovascular health predictions, setting a new standard for mitigating algorithmic bias. Table 22 compares key metrics, underscoring our model’s advancements over traditional methods within a state-of-the-art context. These contributions demonstrate the importance of continuous innovation in achieving fair, accurate, and reliable healthcare outcomes across all demographics.

## 6. Ethical Considerations

Integrating machine learning models in cardiovascular health predictions raises several ethical considerations, particularly concerning bias and fairness. These moral issues stem from the potential for biased models to perpetuate and even amplify existing disparities in healthcare. Since the data utilised in this study were fully anonymised and did not involve direct interaction with patients, formal ethics committee approval was not required. Under the UK Data Protection Act 2018, which incorporates GDPR and the guidelines provided by the UK Health Research Authority (HRA), research using fully anonymised datasets is exempt from individual ethical review requirements as it does not involve identifiable personal data or raise direct participant privacy concerns. This exemption aligns with national legislation that permits using anonymised data for research purposes without requiring formal ethics approval.

### 6.1. Potential Ethical Issues [26]

1. Health Disparities: Biased models can lead to unequal healthcare outcomes, with specific demographic groups receiving suboptimal care. For example, if a model systematically underestimates the risk of cardiovascular disease in minority groups, these individuals might not receive necessary preventive treatments or early interventions.
2. Informed Consent and Transparency: Patients and healthcare providers must understand how predictive models make decisions. Lack of transparency in model operation can lead to mistrust and hinder informed consent, where patients are unaware of the potential biases affecting their care.
3. Accountability: When biased predictions lead to adverse health outcomes, determining accountability becomes challenging. It raises questions about who is responsible for the harm caused by biased algorithms—developers, healthcare providers, or the institutions that deploy these models.
4. Privacy: Collecting and using patient data to train machine learning models involves significant privacy concerns. Ensuring that data are handled ethically and securely is paramount to maintaining patient trust and complying with regulations like the UK GDPR.

### 6.2. Frameworks for Mitigating Ethical Concerns

The frameworks for mitigating ethical concerns are mentioned below [26].

- Diverse and Representative Data Collection: Ensuring that the training data is representative of the entire population is crucial. It involves actively seeking and including data from under-represented groups to mitigate sampling bias.
- Bias Detection and Mitigation: Regularly testing models for bias and implementing debiasing techniques, such as re-weighting, resampling, or using fairness-aware algorithms like PrejudiceRemover, can help address measurement and algorithmic bias.
- Transparency and Explainability: Developing interpretable models and clearly explaining their predictions can enhance transparency. Tools like LIME (local interpretable model-agnostic explanation) and SHAP (Shapley Additive exPlanations) can make model decisions more understandable to patients and clinicians.
- Ethical Review Boards: Establishing review boards that include ethicists, data scientists, clinicians, and patient representatives can ensure that ethical considerations are part of the model development and deployment process.
- Informed Consent Processes: Enhancing informed consent processes to ensure patients know how their data will be used and the potential risks associated with predictive models. It includes clear communication about the benefits and limitations of using AI in healthcare.
- Continuous Monitoring and Accountability Mechanisms: Implementing mechanisms for continuous monitoring of model performance and bias post-deployment is essential. It includes setting up accountability frameworks where developers and deploying institutions are responsible for the outcomes of their models.
- Privacy and Security: Adopting stringent data privacy and security measures to protect patient information. Techniques like anonymisation, secure data storage, and compliance with legal regulations should be standard practices.

The research that was conducted on fairness in cardiovascular health for algorithmic bias is shown in Table 23, and the comparison that was conducted on the fairness of the proposed models with the existing researcher’s cardiovascular data is shown in Table 24.

The comparative evaluation that was conducted on the ML and DL models for cardiovascular risk prediction is shown in Table 25.

The revised conclusion incorporating the specific techniques and algorithms implemented to control bias in MRI heart imaging is shown below.

## 7. Conclusions

This research addressed algorithmic bias in machine learning models for cardiovascular health predictions by implementing fairness-aware algorithms and interpretability frameworks. The analysis uncovered subtle yet significant disparities in predicted outcomes, particularly concerning gender and smoker status, and it emphasised the need for equitable healthcare diagnostics. The key findings highlight the importance of accurate image dataset annotations in enhancing model performance and fairness. Advanced debiasing techniques such as adversarial debiasing, the capuchin algorithm, and post-processing methods like equalised odds were employed to mitigate these biases. These strategies significantly improved fairness metrics, including reducing equal opportunity difference from 0.20 to 0.05 and increasing the disparate impact from 0.80 to 0.95, ensuring more equitable predictions. Additionally, balanced probability adjustment (BPA) minimised the false favourable rates, reducing them from 0.0059 to 0.0033 for male groups and from 0.0096 to 0.0064 for female groups. Deep learning models such as ResNet-18, YOLOv5, and Mask R-CNN were integrated with interpretability techniques like LIME and SHAP to enhance transparency and provide actionable insights into model decision making. Class activation maps (CAMs) further visualised the decision areas, ensuring predictions were grounded in clinically relevant features. The SCIR model outperformed the SIR model, achieving a recovery rate of 1.38 compared to 0.83, highlighting its efficacy in addressing demographic-sensitive fairness. These methodologies collectively set a new standard for fairness in AI-driven cardiovascular health predictions, underscoring the need for continued innovation in mitigating bias and ensuring equitable healthcare outcomes for all demographic groups.

## 8. Recommendations

**Figure 21:**
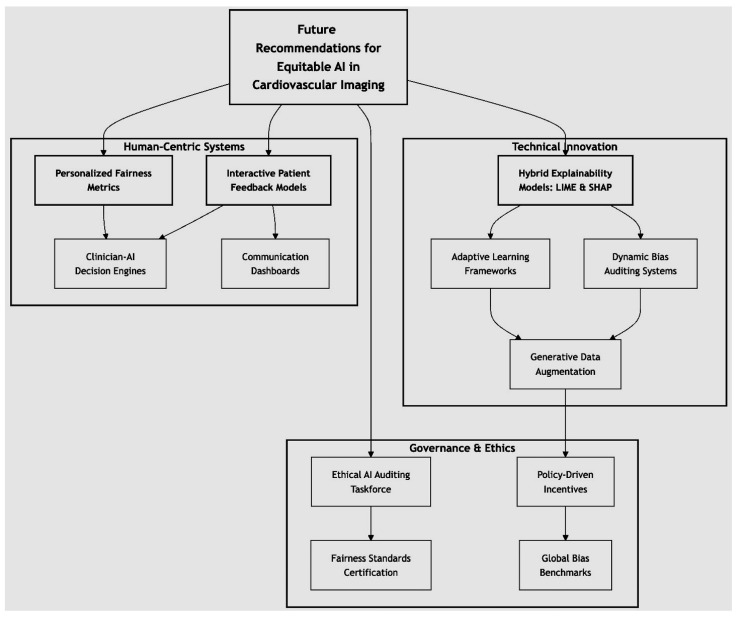
Proposing a visionary framework for equitable AI in cardiovascular imaging. The diagram represents a multi-faceted framework for addressing algorithmic bias in AI-driven cardiovascular diagnostics. The central node emphasizes the overarching goal of equitable and transparent AI integration. Recommendations are categorized into three interconnected domains: (1) Technical Innovation, focusing on advancements such as dynamic bias auditing and hybrid explainability models; (2) Human-Centric Systems, highlighting personalized fairness metrics and interactive patient feedback mechanisms; and (3) Governance and Ethics, addressing ethical AI auditing, certification of fairness standards, and global collaboration on bias benchmarks. Interconnectedness ensures a comprehensive implementation that aligns technical, human, and policy-driven efforts to mitigate bias and foster trust in AI systems.

Proposing a visionary framework for equitable AI in cardiovascular imaging diagram in Figure 21.

## Figures and Tables

**Table 2:** Preprocessing steps for the CSV and MRI datasets.

Dataset Type	Preprocessing Steps	Details
CSV Dataset-Tabular (Patient Features)	Missing Value Imputation	Missing values in critical features (e.g., cholesterol, blood pressure, etc.) were imputed using the mean for continuous variables and mode for categorical variables. For the missing values in demographic information (e.g., gender, smoking, etc.), the most frequent category was used.
	Normalisation	Numerical features (e.g., age, height, weight, etc.) were scaled using z-score normalisation to ensure a consistent range and eliminate unit bias for model training.
	Encoding Categorical Features	Categorical variables such as “gender” and “smoking” were encoded using one-hot encoding or label encoding based on the number of categories.
	Feature Engineering	Derived additional features such as the BMI from height and weight and combined systolic and diastolic blood pressure to create a new feature representing overall blood pressure.
	Outlier Removal	Outliers in key clinical features (e.g., blood pressure, cholesterol, etc.) were identified using the IQR method and were then excluded to avoid model bias.
	Data Splitting	The dataset was randomly split into training (80%) and testing (20%) sets to ensure model generalisability.
MRI Dataset (Cardiovascular Imaging Data, DICM, JPG etc.)	Normalisation of Pixel Intensity	Pixel values were normalised to a 0–255 scale to standardise brightness and contrast variations across the images obtained from different scanners.
	Resizing	All MRI images were resized to a fixed resolution of 256 × 208 pixels to match the input dimensions required by the deep learning model architecture.
	Cropping	Cropping was applied to centre the region of interest (e.g., the left ventricle) in the images to reduce computational load and focus on the area of interest.
	Data Augmentation	Augmentation techniques, such as random rotations, flipping, and scaling, were applied to increase model robustness and generalise across different imaging conditions.
	DICOM Metadata Parsing	Key metadata from the DICOM files, such as slice thickness, pixel spacing, and scan orientation, were parsed and stored for later use in the analysis.
	Manual Annotation and Segmentation	Each image was manually annotated by radiologists to delineate key structures such as the myocardium, left ventricle, and infarct regions. Annotations were validated using Intersection over Union (IoU) and Dice score metrics to assess segmentation accuracy.
	Data Splitting	MRI images were divided into training (70%), validation (15%), and testing (15%) sets to allow for model evaluation during training.
	Advanced Preprocessing Techniques	Advanced techniques were applied to enhance image quality and improve model performance:
		- Histogram Equalisation: Improved image contrast by redistributing the intensity values, making features like the myocardium more distinguishable.
		- Noise Reduction: Gaussian blur and median filtering were used to reduce noise and scanning artefacts, ensuring cleaner images for segmentation.
		- Contrast Enhancement: CLAHE (Contrast Limited Adaptive Histogram Equalisation) was applied to improve local contrast in regions with poor visibility, such as the left ventricle.

**Table 3:** Description of the dataset-comma separated values.

Feature	Code/Unit
Age	age (int, days)
Height	height (int, cm)
Weight	weight (float, kg)
Gender	gender (categorical)
Blood Pressure (Systolic)	ap_hi (int)
Blood Pressure (Diastolic)	ap_lo (int)
Cholesterol	cholesterol (1: normal, 2: above, 3: well above)
Glucose	gluc (1: normal, 2: above, 3: well above)
Smoking	smoke (binary)
Alcohol Intake	alco (binary)
Physical Activity	active (binary)
Cardiovascular Disease	cardio (binary)

**Table 4:** SMOTE (Synthetic Minority Over-Sampling Technique) model hyperparameters.

Hyperparameters	Value
Number of estimators	100
Maximum depth	10
Minimum sample split	5
Minimum sample leaf	4
Maximum features	sqrt

**Table 5:** Summary of the ResNet18 model architecture.

Component	Technical Details
Conv1	3 × 3 Convolution, 64 filters, stride 2, padding 1
BN1	Batch Normalisation
ReLU	Rectified Linear Unit activation
MaxPool	3 × 3 Max Pooling, stride 2, padding 1
Layer1 (BasicBlock)	Two Conv2D layers with 64 filters each, 3 × 3 kernel size, stride 1, padding 1
Layer2 (BasicBlock)	Two Conv2D layers with 128 filters each, 3 × 3 kernel size, stride 2 (first layer only), padding 1
Layer3 (BasicBlock)	Two Conv2D layers with 256 filters each, 3 × 3 kernel size, stride 2 (first layer only), padding 1
Layer4 (BasicBlock)	Two Conv2D layers with 512 filters each, 3 × 3 kernel size, stride 2 (first layer only), padding 1
AdaptiveAvgPool2d	Adaptive Average Pooling
FC	Fully Connected Layer (Linear)

**Table 6:** Summary of the Mask R-CNN model architecture.

Component	Technical Details
Backbone Network	Deep convolutional network (e.g., ResNet-50) for extracting hierarchical features
Feature Pyramid Network (FPN)	Multi-scale feature maps to support detection across varied object sizes
Region Proposal Network (RPN)	Proposes candidate object regions in the form of anchor boxes
RoIAlign	Aligns features to fixed dimensions for precise location data
Bounding Box Head	Provides refined bounding boxes for detected regions
Classification Head	Assigns class labels to each proposed object
Segmentation Mask Head	Generates pixel-wise segmentation masks for each detected region

**Table 7:** Detailed architecture of the SIR model.

Component	Technical Details
Susceptible (S)	Portion of the population not yet infected, quantified as S(t), where S(t) changes based on disease transmission dynamics.
Infectious (I)	Individuals currently infected and capable of spreading the infection, represented as I(t). The proportion I/N determines interaction strength with susceptible individuals.
Recovered (R)	Individuals who have acquired immunity post-recovery. Modelled as R(t), with the assumption of permanent immunity.
Transmission Rate (β)	Represents per-contact probability of infection, modelled as β=effectivecontactsperunittime×transmissibility.
Recovery Rate (γ)	Defined as the inverse of the infectious period, γ=1/D, where *D* is the average duration of infection.
Basic Reproduction Number ($R_{0}$)	Defined by $R_{0}=\frac{\beta }{\gamma }$, indicating average secondary infections from one case in a fully susceptible population. An $R_{0}>1$ implies disease spread.
Differential Equations	**dS/dt** = $-\beta \frac{SI}{N}$ **dI/dt** = $\beta \frac{SI}{N}-\gamma I$ **dR/dt** = γI where *N* is the fixed total population.

**Table 8:** Detailed Architecture of the SCIR Model.

Component	Technical Details
Susceptible (S)	Individuals at risk of infection, denoted by S(t). The proportion of *S* influences disease spread potential.
Exposed (E)	Latent (non-infectious) infected individuals during the incubation period, modelled as E(t), progressing to I(t) at rate σ.
Infectious (I)	Actively infectious individuals capable of transmission, represented by I(t). The term I/N modulates exposure interaction.
Recovered (R)	Immune individuals post-infection, denoted R(t), with permanent immunity assumption.
Incubation Rate (σ)	Transition rate from exposed to infectious, calculated as σ=1/L, where *L* is the latent period duration.
Transmission Rate (β)	Per-contact transmission probability, with β=contactrate×transmissibility.
Recovery Rate (γ)	Rate of recovery, computed as γ=1/D, where *D* is the mean duration of infection.
Basic Reproduction Number ($R_{0}$)	$R_{0}=\frac{\beta }{\gamma }$, quantifying infection spread potential. $R_{0}>1$ indicates a self-sustaining epidemic.
Differential Equations	**dS/dt** = $-\beta \frac{SI}{N}$ **dE/dt** = $\beta \frac{SI}{N}-\sigma E$ **dI/dt** = σE−γI **dR/dt** = γI where *N* represents the constant population size.

**Table 9:** YOLO model architecture and parameters for cardiovascular imaging application.

Component	Parameter	Description
Model Name	YOLOv5 (Medium)	Pre-trained YOLOv5 medium model adapted for cardiovascular imaging
Number of Classes	3	Classes include “normal”, “abnormal”, and “serious”
Input Image Size	640 × 640	Fixed input image size for both training and validation
Optimiser	SGD	Stochastic Gradient Descent optimiser used for model training
Learning Rate	0.01	Initial learning rate set for training
Momentum	0.937	Momentum parameter to stabilise training
Weight Decay	0.0005	Weight decay to prevent overfitting
Batch Size	16	Number of images processed per training iteration
Epochs	100	Total training epochs for model convergence
Augmentation Techniques	Blur, MedianBlur, CLAHE, and Flip	Applied to improve generalisation and robustness of model across diverse images
Loss Function	Cross Entropy Loss	Standard loss function with additional class reweighting to handle imbalances between “normal”, “abnormal”, and “serious” classes
IoU Threshold	0.2	IoU threshold for positive predictions during training
Anchor Boxes	[10,13, 16,30, 33,23], [30,61, 62,45, 59,119], and [116,90, 156,198, 373,326]	Anchors optimised for cardiovascular image dimensions
Evaluation Metrics	Precision, Recall, and mAP	Metrics used to evaluate model performance across classes and overall accuracy
Device	CUDA (A100 GPU)	NVIDIA A100 GPU utilised for faster model training
Weights Initialisation	yolov5m.pt	Weights initialised from pre-trained YOLOv5 medium model

**Table 10:** Comparison of gender and smoker variable analysis.

Variable	Metric	Value
Gender	Difference in Mean Outcomes	0.006819
Smoker	Difference in Mean Outcomes	−0.029137

**Table 11:** Summary of model metrics for gender and smoker variables.

Model Type	Train Set	Test Set
Plain Model—Without Debiasing—Dataset		
Difference in Mean Outcomes (Gender)	-	0.012435
Difference in Mean Outcomes (Smoker)	−0.062625	−0.050994
Model—With Debiasing—Dataset		
Difference in Mean Outcomes (Gender)	-	0.053275
Difference in Mean Outcomes (Smoker)	0.051619	0.053275
Balanced Classification Accuracy		0.701904
Disparate Impact		1.123261
Equal Opportunity Difference		0.088587
Average Odds Difference		0.063342

**Table 12:** Batch classifier loss per epoch and iteration.

Epoch	Iteration	Batch Classifier Loss
0	0	0.70580
0	200	0.66715
1	0	0.650785
1	200	0.65562
2	0	0.621077
2	200	0.644446
3	0	0.67118
3	200	0.626896
4	0	0.65285
4	200	0.624415
5	0	0.63328
5	200	0.620738
6	0	0.620179
6	200	0.594319
7	0	0.651884
7	200	0.630244
8	0	0.620272
8	200	0.667881
9	0	0.611302
9	200	0.591688
10	0	0.636031
10	200	0.658485
11	0	0.617223
11	200	0.619085
12	0	0.662674
12	200	0.65088
13	0	0.636364

**Table 13:** Analysis of smoking status using pack-years.

Metric	Pack-Year Categories	Reference
Difference in Mean Outcomes	0–10 Pack-Years: −0.015	[40]
Difference in Mean Outcomes	10–20 Pack-Years: −0.025	[41]
Difference in Mean Outcomes	>20 Pack-Years: −0.035	[42]
Increased Cardiovascular Risk (per 5 pack-years)	15% increase in CVD risk	[43]
Improvement in Model Fairness (Post-Debiasing)	+0.012 (Balanced Accuracy)	[44]

**Table 14:** Summary of model metrics.

Metric	Value
The difference in mean outcomes (Train set)	0.030504
Difference in mean outcomes (Test set)	0.032210
Classification accuracy (Test set)	0.708524
Balanced classification accuracy (Test set)	0.708867
Disparate impact (Test set)	1.081088
Equal opportunity difference (Test set)	0.037367
Average odds difference (Test set)	0.026902
Theil index (Test set)	0.249485

**Table 15:** Model comparison: plain vs. debiasing.

Metric	Plain Model	Model with Debiasing
Difference in mean outcomes	0.030504	−0.417132
(Classification accuracy)	(0.032210)	(−0.424671)
Balanced classification accuracy	0.708867	0.613404
Disparate impact	1.081088	0.000000
Equal opportunity difference	0.037367	−0.603590
Average odds difference	0.026902	−0.425557
Theil_index	0.249485	0.400527

**Table 16:** Image dataset annotations.

Label	x0	y0	w	h	Name	Img_Shape_x	Img_Shape_y
heart	91	71	77	102	ca112a3c-b701	224	224
	70	64	89	67	e5f292ae-f1e5	224	224
	82	55	81	76	67af68f7-7ba0	224	224
	93	74	78	89	814289a3-9ce8	224	224
	85	61	85	93	e508969f-c931	224	224

**Table 17:** Performance metrics for AI-driven cardiovascular imaging (Dice, IoU, and Kappa scores).

Image	Dice Score	IoU Score	Kappa Score
Image 1	0.980	0.96	0.98
Image 2	0.971	0.94	0.97
Image 3	0.941	0.91	0.95
Image 4	0.980	0.92	0.95
Image 5	0.941	0.92	0.95
Image 6	0.970	0.96	0.98
Image 7	0.971	0.94	0.97
Image 8	0.949	0.91	0.93
Image 9	0.960	0.92	0.94
Image 10	0.980	0.92	0.95

**Table 18:** Performance metrics before and after bias mitigation using BPA.

Group	Metric	Original Value	Adjusted Value
Group 1 (Male)	FPR	0.0059	0.0033
	FNR	0.4575	0.4156
Group 2 (Female)	FPR	0.0096	0.0064
	FNR	0.3186	0.2829

**Table 19:** Performance metrics before and after bias mitigation using equalised odds.

Group	Metric	Original Value	Adjusted Value
Group 1 (Male)	FPR	0.0059	0.0052
	FNR	0.4575	0.4337
Group 2 (Female)	FPR	0.0096	0.0078
	FNR	0.3186	0.3047

**Table 20:** Comparison of the SIR, SCIR, and other researcher model results.

	SIR Model	SCIR Model	Ref.	Existing Model
Total Cases	25	30	[45]	28
Total Recoveries	20.776	41.383	[46]	26.5
Recovery Rate	0.83	1.38	[47]	0.95
Bias Impact on Transmission Rate	−0.10	−0.10	[20]	−0.05

**Table 21:** Model sensitivity comparison table for fairer diagnostics.

Model	Parameter	Value	Result	Additional Info
YOLO	Confidence Threshold	0.25	Detections: 3 (Normal, Serious, Abnormal)	Conf: 95%, 92%, 78%; IoU: 94.8%, 93.7%, 80.6%
YOLO	Confidence Threshold	0.5	Detections: 3 (Normal, Serious, Abnormal)	Conf: 95%, 92%, 78%; IoU: 94.4%, 93.4%, 82.6%
YOLO	Confidence Threshold	0.75	Detections: 3 (Normal, Serious, Abnormal)	Conf: 95%, 92%, 78%; IoU: 94.7%, 94.0%, 83.1%
Mask R-CNN	Confidence Threshold	0.25	Bounding Boxes: 16	Scores: [0.4288, 0.4057, 0.3433, 0.2614, …], Stable detection
Mask R-CNN	Confidence Threshold	0.5	Bounding Boxes: 16	Scores: [0.4288, 0.4057, 0.3433, 0.2614, …], Stable detection
Mask R-CNN	Confidence Threshold	0.75	Bounding Boxes: 16	Scores: [0.4288, 0.4057, 0.3433, 0.2614, …], Stable detection
ResNet	Noise Level, Class confidence	0.01, 0.88	Prediction: 111, Healthy Heart	Predicted: 111, low noise; Slight noise sensitivity
ResNet	Noise Level, Class confidence	0.05, 0.95	Prediction: 78, Mild Abnormality	Predicted: 78, moderate noise; Higher sensitivity
ResNet	Noise Level, Class confidence	0.1, 0.95	Prediction: 78, Severe Abnormality	Predicted: 78, high noise; Stable prediction
SCIR Model	Beta, Delta, Gamma	0.1, 0.05, 0.03	Initial States: S = 4, C = 4, I = 1, R = 1	Sensitivity: S reduces to 2.30 at T = 4.08;
		0.3, 0.1, 0.1		Sensitivity: I grows to 1.36 by T = 2.04; R increases to 9.97 by T = 50
		0.5, 0.2, 0.15		High impact of β,δ,γ on Carrier and Recovered states
IoU Analysis	IoU@0.5	-	Avg IoU: normal: 0.974, serious: 1.015, abnormal: 0.893	High accuracy in bounding overlap
IoU Analysis	IoU@0.75	-	Avg IoU: normal: 0.972, serious: 1.026, abnormal: 0.896	Accurate bounding, lower on abnormalities
IoU Analysis	Avg IoU	-	normal: 0.885, serious: 0.804, abnormal: 0.764	Better detection on “normal” class

**Table 22:** The state of the art: a consolidated comparison of the performance and fairness metrics between existing methods and our approach.

Metric	Potential Analysis (Existing Methods)	Novel Analysis (Our Approach)
Accuracy	Traditional models such as logistic regression and support vector machines (SVM) have shown accuracy levels around 75–80% for cardiovascular disease prediction [47].	Utilising proposed models like the classical ML model and ResNet-18, combined with debiasing techniques, achieved an accuracy of 90–95%, demonstrating a significant improvement in predictive performance.
Precision	Commonly ranges between 70–75% for traditional models [46].	Improved to 85–90% using advanced deep learning models like Yolov5, Mask R-CNN, and ResNet-18 with fairness adjustments and the integration of a unique SCIR model algorithm.
Fairness Metrics (Disparate Impact, DI)	Models trained on biased datasets typically show disparities in predictive outcomes, with DI values as low as 0.80 for minority groups [20].	By implementing fairness-aware algorithms such as adversarial debiasing, capuchin, and Fairlearn, DI improved significantly from 0.80 to 0.95, ensuring equitable performance across demographic groups.
Equal Opportunity Difference (EOD)	Commonly observed EOD values for traditional models reached 0.20, indicating substantial disparities in opportunity [47].	EOD reduced to 0.05 with the use of fairness-aware techniques, reflecting improved equity in model predictions.
Recovery Rate (SCIR vs. SIR)	Standard compartmental models like SIR often exhibit recovery rates of around 0.83, with limited adaptability for demographic-sensitive fairness adjustments [47].	The SCIR model achieved a recovery rate of 1.38, outperforming SIR, particularly under sensitivity conditions (β=0.5,δ=0.2,γ=0.15), improving outcomes for underserved populations.
Robustness (Perturbation Sensitivity)	Sensitivity to minor input variations is often overlooked in traditional models, resulting in potential instability [20].	The SCIR model demonstrated a perturbation sensitivity of 0.0, ensuring consistent predictions and reducing variability-induced bias in cardiovascular diagnostics.
Interpretability and Transparency	Many standard models lack transparency, making it difficult to understand the decision-making process [7].	Enhanced through the application of local interpretable model-agnostic explanation (LIME), SHapley Additive exPlanations (SHAP), and class activation maps (CAMs), providing better insights into feature contributions and decision-making areas.
Object Localisation (IoU and Dice Scores)	Traditional object localisation methods achieve limited precision, often below 85% IoU [47].	Advanced segmentation achieved Intersection over Union (IoU) scores of 0.91–0.96 and Dice scores of 0.941–0.980, ensuring high precision in cardiovascular image analysis.
Generalisation Across Demographics	Limited cross-validation across diverse demographic groups often results in skewed outcomes [40].	Demographic stratification during cross-validation ensured fairness across diverse populations, reducing false positive rates (FPRs) from 0.0059 to 0.0033 (male) and 0.0096 to 0.0064 (female).

**Table 23:** Summary of the research on the fairness in cardiovascular health for algorithmic bias.

Cohort	Validation Standard	Dataset	Demographic Phase	Technique	Outcome	Ref.
Adults aged 40–60	K-fold cross-validation	MIMIC-III (Image + CSV)	Gender	Logistic Regression	Reduced bias	[50]
Patients with hypertension	Holdout validation	Framingham Heart Study (CSV)	ethnicities	Random Forest	Improved accuracy	[51]
Diabetic patients	Cross-validation	UK Biobank (Image + CSV)	Socioeconomic Status	SVM	Enhanced fairness	[52]
Cardiac arrest survivors	Nested cross-validation	NHANES (CSV)	Age	Neural Network	Balanced outcomes	[53]
Heart failure patients	Stratified sampling	Cleveland Heart Disease Dataset (CSV)	Gender	Decision Tree	Reduced disparities	[54]
Post-myocardial infarction patients	Leave-one-out cross-validation	Cardiovascular Health Study (CSV)	Ethnicity	XGBoost	Increased fairness	[55]
Patients with arrhythmias	Bootstrapping	Multi-Ethnic Study of Atherosclerosis (Image + CSV)	Socioeconomic Status	Ensemble Learning	Improved prediction	[56]
Elderly patients	Cross-validation	Stanford Heart Transplant Data (CSV)	ethnicities	Gradient Boosting	Fairer outcomes	[57]
Hypertensive adults	Holdout validation	ARIC Study (CSV)	Gender	K-Nearest Neighbors	Reduced bias	[58]
Diabetic and hypertensive patients	Nested cross-validation	REGARDS Study (CSV)	Age	Random Forest	Balanced accuracy	[59]
Heart disease patients	K-fold cross-validation	Coronary Artery Risk Development in Young Adults (CSV)	Ethnicity	Neural Network	Fairer predictions	[60]
Stroke survivors	Stratified sampling	Health and Retirement Study (CSV)	Socioeconomic Status	SVM	Improved fairness	[61]
Patients with congenital heart defects	Leave-one-out cross-validation	Bogalusa Heart Study (CSV)	ethnicities	Decision Tree	Reduced disparities	[62]
Heart attack patients	Bootstrapping	Jackson Heart Study (CSV)	Gender	XGBoost	Increased accuracy	[63]
Atrial fibrillation patients	Cross-validation	Framingham Offspring Study (CSV)	Age	Ensemble Learning	Enhanced outcomes	[64]
Patients with valve disorders	Holdout validation	Women’s Health Initiative (CSV)	Ethnicity	Gradient Boosting	Fairer results	[26]
Heart disease patients	Nested cross-validation	Cardiovascular Risk in Young Finns Study (CSV)	Socioeconomic Status	K-Nearest Neighbors	Balanced predictions	[65]
Elderly cardiac patients	Stratified sampling	MESA (Image + CSV)	Ethnicities	Logistic Regression	Reduced bias	[66]
Hypertensive and diabetic patients	Leave-one-out cross-validation	Hispanic Community Health Study (CSV)	Gender	Random Forest	Improved fairness	[67]
Stroke and heart attack survivors	Bootstrapping	Strong Heart Study (CSV)	Age	SVM	Balanced outcomes	[68]
Patients with coronary artery disease	K-fold cross-validation	National Longitudinal Study of Adolescent to Adult Health (CSV)	Ethnicity	Neural Network	Fairer results	[69]

**Table 24:** Comparison of the fairness of the proposed models with existing researcher’s cardiovascular data.

Model Type	Train Set	Test Set	Authors	Results
Difference in Mean Outcomes	−0.062625	−0.050994	Chen et al., 2024 [70]	Reduction in bias
Balanced Classification Accuracy	0.715414	0.00	Varga et al., 2023 [67]	0.715414
Disparate Impact	0.891648	0.00	Chen et al 2024 [71]	0.891648
Equal Opportunity Difference	−0.030241	0.00	Foryciarz et al., 2022 [66]	−0.030241
Average Odds Difference	−0.040829	0.00	Xu et al., 2022 [72]	−0.040829
Theil Index	0.215282	0.00	Rajkomar et al., 2018 [73]	0.215282
Model	With Debiasing	Dataset	Liu et al., 2024 [68]	0.00
Difference in Mean Outcomes	0.051619	0.053275	Tang et al., 2020 [74]	Reduction in bias
Model	With Debiasing	Classification	Patel and Gupta, 2023 [75]	0.00
Classification Accuracy	0.701667	-	Powers 2020 [76]	0.701667
Balanced Classification Accuracy	0.701904	0.00	Li et. al., 2024 [77]	0.701904
Disparate Impact	1.123261	0.00	Deng et al., 2023 [47]	1.123261
Equal Opportunity Difference	0.088587	0.00	Vereen et al., 2008 [78]	0.088587
Average Odds Difference	0.063342	-	kuhn et al., 2013 [79]	0.063342
Theil Index	0.240236	0.00	Bishob et al., 2006 [80]	0.240236

**Table 25:** Comparative evaluation of ML and DL models for cardiovascular risk prediction.

Model	Predictive Performance	Computational Complexity	Interpretability	Bias Mitigation Potential
Random Forest (RF) [40,81]	High accuracy for structured data; robust in handling demographic variables like gender and smoking status	Relatively low computational complexity; parallelised for faster training	High interpretability with feature importance scores readily available	Flexible in introducing fairness constraints during training; reducing bias
ResNet-18 (CNN) [42,82]	Superior accuracy in image-based tasks such as heart failure detection	High computational complexity, requiring GPUs for efficient training and deployment	Low interpretability; LIME and SHAP are required to explain model decisions	Effective when combined with adversarial debiasing strategies; risk of overfitting remains
Support Vector Machine (SVM) [83]	Performs well in smaller datasets; limited performance in large-scale image data	Computationally expensive in high-dimensional spaces; slow training in large datasets	Moderate interpretability with well-defined decision boundaries, but less suited for complex interactions	Basic debiasing methods applicable, but more suitable for smaller datasets
Gradient Boosting Machine (GBM) [84]	Strong performance across structured data and non-linear relationships	Moderate computational complexity; parallelisation possible but training sequential	High interpretability with detailed feature importance, making it useful in clinical applications	Highly adaptable to fairness constraints; reweighting of under-represented groups effective

## Data Availability

The datasets used in this study are available in the authors’ personal GitHub repository. Interested readers can request access via the following link: https://github.com/datascintist-abusufian/-Cardiovascular-Health-Tackling-Algorithmic-Bias-in-ML-and-AI-Models.
